# Phonon Engineering of Micro‐ and Nanophononic Crystals and Acoustic Metamaterials: A Review

**DOI:** 10.1002/smsc.202200052

**Published:** 2022-11-10

**Authors:** Jihong Ma

**Affiliations:** ^1^ Department of Mechanical Engineering University of Vermont Burlington VT 05405 USA; ^2^ Materials Science Program University of Vermont Burlington VT 05405 USA

**Keywords:** acoustic metamaterials, micro- and nanoscales, numerical simulations, phononic crystals, phonons, theoretical methods, thermal management

## Abstract

Phononic crystals (PnCs) and acoustic metamaterials (AMMs) are artificially architected materials endowed with the capabilities of wave manipulation. This review specifically covers the theoretical fundamentals and recent development of PnCs and AMMs at the micro‐ and nanoscales due to their potential significance in modern electronic applications. Similarities and differences in phonon transport at these two scales are discussed. Research gaps between the macro‐ and small‐scale PnC and AMM development are identified from a theoretical point of view. A future outlook is also presented to propose possible applications of micro‐ and nano‐PnCs and AMMs, including advancing quantum information processing technologies, such as quantum computing.

## Graphical abstract

Phononic crystals and acoustic metamaterials with phonon wave manipulation capabilities have attracted considerable attention. This review summarizes the progress and identifies gaps in phonon engineering at the micro‐ and nanoscales using phononic crystals and metamaterials due to their potential significance in modern electronic applications. An outlook is presented to propose advanced applications, such as quantum computing.

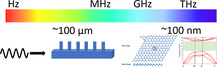

## 1 Introduction

In condensed matter physics, the motion of individual atoms in a crystal lattice can be represented as an ensemble of excitation, each of which has a well‐defined energy and crystal momentum. When quantized, these ensembles of excitation are referred to as phonons, that is, lattice vibrations, with a vibration frequency in THz. Phonons carry atomic vibration energy as they propagate through crystals and have a significant impact on many physical properties, including specific heat, thermal and electrical conductivity, melting and glass transition temperatures, and thermal expansion. Thus, understanding phonon transport mechanisms in nanostructures is essential in phonon propagation manipulation in lattices in order to achieve desirable phononic or even a broader range of physical properties. Depending on the length scale of interest, phonons can be treated as both waves and particles, that is, wave–particle duality. Different treatments of phonons call for various levels of theories that can be used in the study of size effect on phonon transport.^[^ ^1^ ^]^

The first introduction of the concept of phonons can be traced back to 1930 by Tamm^[^ ^2^ ^]^ and was subsequently studied by Frenkel.^[^ ^3^ ^]^ The word phonon originates from the Greek word ϕωvη, which means “voice”. Indeed, long‐wavelength phonons within the audible frequency range (from 20 Hz to 20 kHz) give rise to sound. Such a low‐frequency range typically requires continuum‐level treatment using classical mechanics theories. Regardless of the different means of solving the problems at the atomistic scale and continuum regime, the fundamental principles of vibration are independent of the length scale. Thus, phonon engineering across all spectra should follow a universal rule, with special considerations taken at the nanoscale, such as phonon–photon/electron‐coupling effect,^[^ ^4^, ^5^ ^]^ phonon scattering,^[^ ^6^ ^]^ and quantum confinement effects.^[^ ^7^, ^8^ ^]^

One effective way to engineer phonon dispersion is via a periodic arrangement of materials or structures, that is, phononic crystals (PnCs) and acoustic metamaterials (AMM). As the wave manipulation capabilities of both PnCs and AMMs are governed by their unit cell characteristics, one may find in the literature that the two are often discussed together.^[^ ^9^, ^10^, ^11^, ^12^ ^]^ Nevertheless, their differences lie in the fact that PnCs are periodic and the sizes of their unit cells are on the order of phonon wavelength, while AMMs do not necessarily need to be periodic, and their unit cell sizes are typically smaller than phonon wavelengths, that is, subwavelength. Note that some researchers name the subwavelength periodic structures as PnCs and consider PnCs as a subgroup of AMMs, which is simply another convention of nomenclature.^[^ ^11^, ^13^ ^]^ In this review, the former convention is adopted. Furthermore, in certain periodic structures, such as a PnC plate, the unit cell size can be both subwavelength (at low frequencies) and comparable with wavelength (at high frequencies) and thus can be classified as both PnCs and AMMs.^[^ ^14^ ^]^

As most PnCs and AMMs discussed here are periodic, it enables the analysis of bulk‐wave propagation using a unit cell analysis in the reciprocal domain. We will present in Section 2 and 3 that a unit‐cell analysis can provide convenience to explore many more interesting wave manipulation capabilities, such as mechanical filtering and wave directionality,^[^ ^15^, ^16^, ^17^, ^18^ ^]^ waveguiding,^[^ ^19^, ^20^, ^21^ ^]^ acoustic cloaking,^[^ ^22^, ^23^ ^]^ and energy trapping.^[^ ^24^, ^25^, ^26^ ^]^ A handful of review articles have extensively summarized recent developments of PnCs and AMMs in general.^[^ ^10^, ^11^, ^27^, ^28^ ^]^ As unit cell characteristics determine the bulk wave propagation, the symmetry and topology of a unit cell can yield many more exotic features, such as the manifestation of bulk characteristics on edges or corners,^[^ ^29^, ^30^, ^31^, ^32^, ^33^ ^]^ also known as the bulk edge correspondence.

Despite a plethora of intriguing routes proposed to engineer phonons using PnCs and AMMs, much of the research in this area has been limited to macroscopic structures. However, many of these wave manipulating approaches can also be helpful in micro‐/nanoelectromechanical systems (M/NEMS) and on‐chip device applications, including energy harvesting, sensing, signal processing, and cloaking. The difficulty lies in the high demand for fabrication precision^[^ ^9^ ^]^ and the increased mechanical complication at the micro‐ and nanoscales. Although with the development of manufacturing technology such difficulty will eventually be conquered, a good understanding of the phonon waves (including both linear and nonlinear behaviors), accurate and efficient theoretical predictions of the phononic behaviors, and an optimized topological design are still critical in achieving desirable PnCs and metamaterials (MMs) at the micro‐ and nanoscales without much trial‐and‐error effort.

In this review, the fundamental principles of phononic wave manipulation will be listed first in Section 2. Then, the size effect on theories, analytical approaches, characteristics, and wave manipulation methods in PnCs and AMMs at the micro‐ and nanoscales are presented in Section 3. The nonconventional PnCs and AMMs, specifically, topological insulators (Tis), at both micro‐ and nanoscales, are subsequently discussed in Section 4. Conclusions of this review and an outlook on this field are laid out in Section 5.

## 2 Fundamental Principles of Phononic Wave Manipulation

### 2.1 Phonon Dispersion Relation

**Figure 1:**
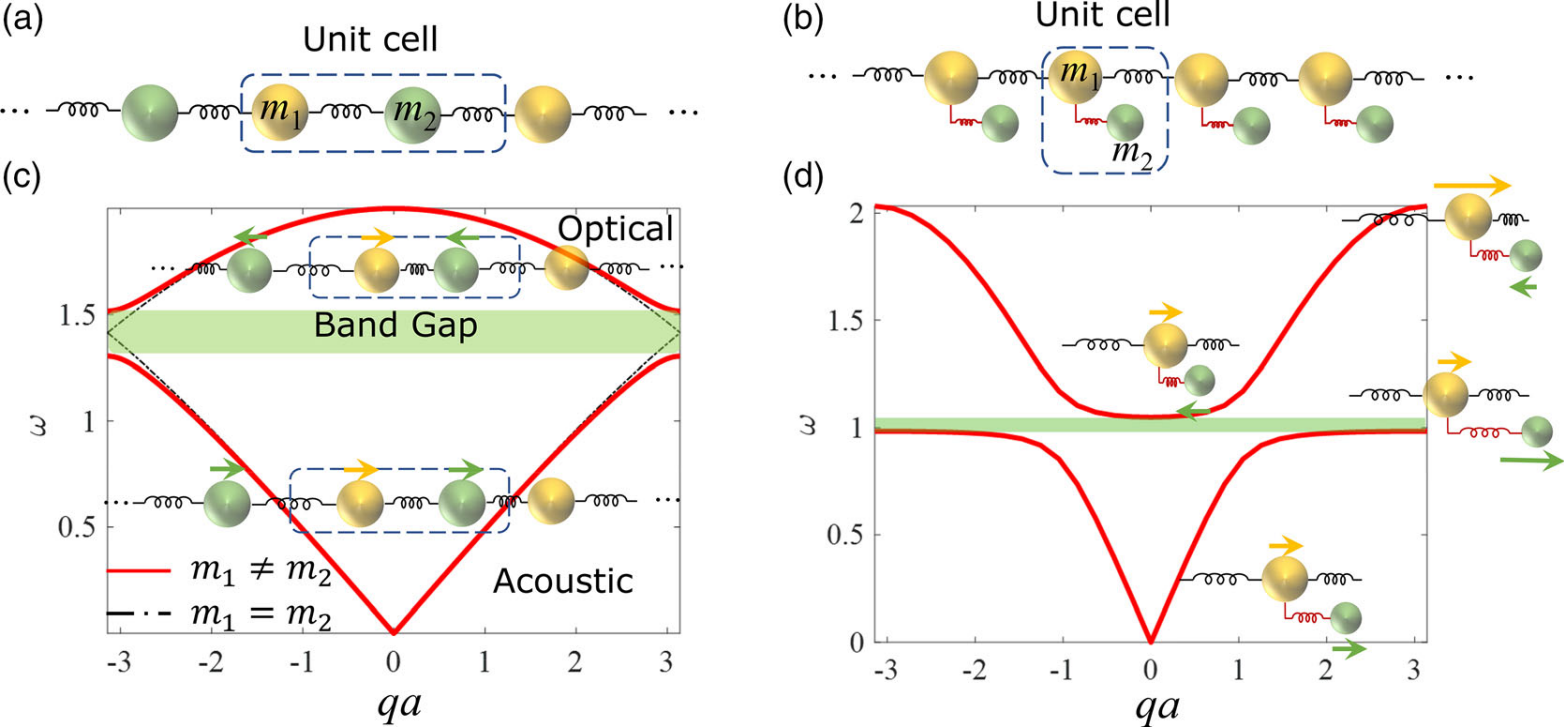
a,b) A 1D spring‐mass system with identical spring constants connecting periodically arranged masses, $m_{1}$ and $m_{2}$ (a) and identical springs and masses, $m_{1}$ (b). Each $m_{1}$ is connected with a resonator with mass $m_{2}$ via a secondary spring (red). c,d) Phonon dispersion relations of a system with $m_{1}$ = $m_{2}$ (black dashed curves) and a diatomic system with $m_{1}$ ≠ $m_{2}$ (red solid curves) with a bandgap in green shade due to Bragg scattering (c) and a system with a low‐frequency bandgap due to local resonance (d). The insets in (c) and (d) show the mode shapes of the unit cells.

Phonons are vibrations of masses or atoms in periodically arranged states of matter. Phonon characteristics, such as the relationship between vibration frequencies, wavelengths, and mode shapes, can be characterized by analyzing phonon dispersion relations determined by spring constants and masses. For example, in a periodic 1D linear spring‐mass system (**Figure** **1**a) with a uniform spring constant, *k* (at the nanoscale, this means all atoms are vibrating around their equilibrium positions, i.e., harmonic oscillations), we can write down the following governing equations of a lattice unit cell consisting of a pair of masses, $m_{1}$ and $m_{2}$, vibrating along the 1D chain (without damping).

(1) $$m_{1}{\ddot{u}}_{1}^{n}=k(u_{2}^{n}-u_{1}^{n})-k(u_{1}^{n}-u_{2}^{n-1})$$

(2) $$m_{2}{\ddot{u}}_{2}^{n}=k(u_{1}^{n+1}-u_{2}^{n})-k(u_{2}^{n}-u_{1}^{n})$$

where displacements of the two masses in the *n*‐th cell are denoted as $u_{1}^{n}$ and $u_{2}^{n}$, respectively, and can be expressed using a planewave solution in combination with Bloch–Floquet^[^ ^34^, ^35^, ^36^ ^]^ periodic boundary conditions (PBCs) (ideally, at the nanoscale, such a planewave can be achieved via monochromatic radiation, such as visible light, X‐rays, neutrons, and electrons, incident upon the crystal).

(3) $$u^{n}(t)=\tilde{u}(q)e^{i\omega t}$$

where *ω* is the vibration frequency, $u^{n}$ are the displacements of the *n*‐th cell with $u^{n}=[u_{1}^{n},u_{2}^{n}]$, and $\tilde{u}(q)=u_{0}e^{-iqa}$, where *q* is the wave number, which is inversely proportional to the wavelength *λ*, that is, q=2π/λ, *a* denotes the lattice constant and $u_{0}$ are displacements within the unit cell. Substituting this expression in Equation (1) and (2) gives

(4) $$[K(q)-\omega^{2}M]\tilde{u}(q)=0$$

where K(q) is the stiffness matrix of the periodic system.

(5) $$K(q)=\left[\begin{array}{cc} 2k & -k-ke^{iqa}\\ k+ke^{-iqa} & 2k \end{array}\right]$$

and ***M*** is the mass matrix

(6) $$M=\left[\begin{array}{cc} m_{1} & 0\\ 0 & m_{2} \end{array}\right]$$

Solving Equation (4) yields the eigensolutions (i.e., eigenfrequencies, *ω*) at each wave number *q*, as shown in Figure 1c.

For a monatomic system with $m_{1}$=$m_{2}$, we can obtain gapless solutions of eigenfrequencies, *ω*, while a bandgap with no eigenfrequencies exist for a diatomic system with $m_{1}$ ≠ $m_{2}$, indicating that vibration waves do not propagate through the system when excited at the frequency within this bandgap. The phonon branches below and above the bandgap are identified as acoustic and optical phonons due to their coherent and out‐of‐phase movements, respectively. The eigenvectors of Equation (4) will inform us of the mode shapes (displacement fields) of the unit cell at each *q* and *ω*, as shown in Figure 1c. Such a bandgap due to the periodicity of material properties is usually referred to as a Bragg bandgap, typically included in the design of PnCs, in which the wavelengths are comparable with the periodic lengths. It is worth noting that the material properties are not limited to lattice mass mismatch; stiffness (spring constant) mismatch and a combination of the two will also result in the same type of bandgap.

Another bandgap‐opening mechanism that is widely studied in AMMs is local resonance. A simplified 1D example is shown in Figure 1b, where each mass $m_{1}$ in the backbone (connected with a spring $k_{1}$) is connected with a secondary mass, $m_{2}$, via a softer spring $k_{2}$, that is, a local resonator. The governing equations in a unit cell then become

(7) $$m_{1}{\ddot{u}}_{1}^{n}=k_{1}(u_{1}^{n+1}-u_{1}^{n})-k_{1}(u_{1}^{n}-u_{1}^{n-1})+k_{2}(u_{2}^{n}-u_{1}^{n})$$

(8) $$m_{2}{\ddot{u}}_{2}^{n}=k_{2}(u_{1}^{n}-u_{2}^{n})$$

The stiffness matrix K(q) then becomes

(9) $$K(q)=\left[\begin{array}{cc} k_{2}-k_{1}(e^{-\mathrm{iqa}}+e^{\mathrm{iqa}}-2) & -k_{2}\\ -k_{2} & k_{2} \end{array}\right]$$

Such a system can also open a low‐frequency bandgap in a subwavelength regime compared with that of Bragg scattering. Band flattening is mostly due to kinetic energy absorption by local resonators, as shown in Figure 1d. Such resonators in a realistic system often exist as pillars, spheres, or stripes supported by a host structure. In a system with both periodicity and resonators, bandgaps will open due to both Bragg scattering and local resonance. However, one usually dominates over the other depending on the local resonator masses and the secondary spring constants when compared with the mass of the main body and the backbone spring constants.^[^ ^37^ ^]^

In addition to bandgaps due to Bragg scattering and local resonance, a third type of bandgap in the super‐wavelength regimes at higher frequencies (above Bragg bandgaps) can be achieved when considering the internal deformation of resonators. Such superwavelength bandgaps are important in engineering thermal transport^[^ ^38^ ^]^ and GHz–THz phonon engineering applications^[^ ^39^ ^]^ at nano‐ and microscales.

For both micro‐ and nanoscale systems, a phonon dispersion analysis would be informative to understand the linear vibration of PnCs and AMMs. For microscale lattices with soft matters as constituents or under large deformation or instability, nonlinearity should also be included in their governing equations to incorporate the nonlinear contribution to the phonon dispersion (discussed in Section 3.1.3). At the nanoscale, such a relation also has implications for thermal transport (discussed in Section 3.2). Although the numerical approaches at the two length scales are nonidentical (i.e., finite‐element analysis [FEA] and atomistic simulations are usually used in micro‐ and nanoscale systems, respectively), all are aimed to solve Equation (4) to obtain the phonon dispersion relation and mode shapes for phonon characteristics analysis.

### 2.2 Wave Propagation Directionality and Velocities

In addition to the ω−q (or ω−λ) relationship revealed in phonon dispersion relations, another important dynamical property of PnCs and MMs with higher dimensionalities is their inherent directionality, which manifests as a frequency‐selective spatial anisotropy of their bulk wave modes. The directionality patterns are dictated by the symmetry of the unit cell and can be acquired via a group velocity ($v_{g}$) calculation at each excitation frequency by taking the spatial derivative of the eigenfrequencies, *ω*, with respect to the wave vector, ***q***, that is, $v_{g}=d\omega /dq$. Hence, the wave propagates in the direction perpendicular to the isofrequency contour.

**Figure 2:**
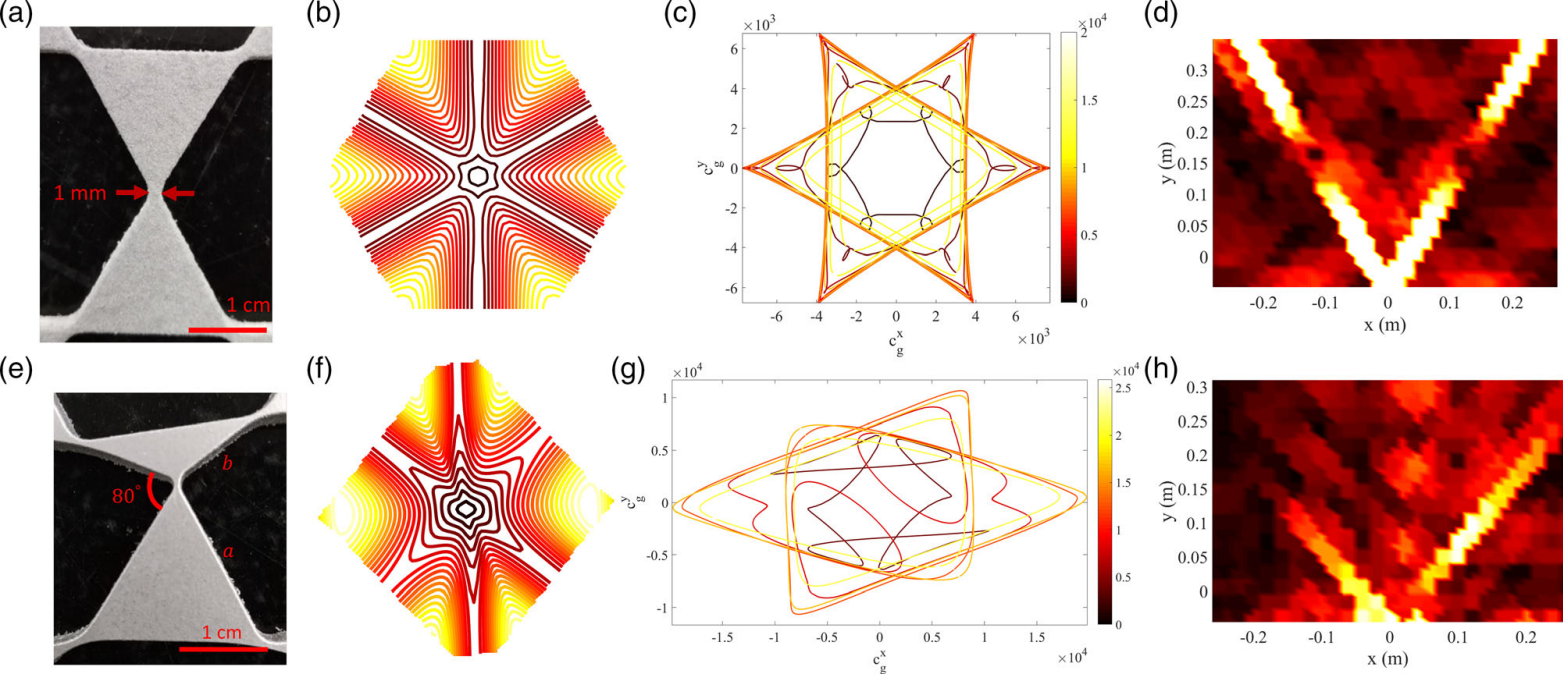
a,e) Experimental specimens of a regular Kagome lattice (a) and a topological Kagome lattice (e). b,f) Isofrequency contours (first phase‐constant surface in the first Brillouin zone) of regular (b) and topological (f) Kagome lattices calculated from FEA. c,g) Group velocity isofrequency contours (frequency increasing from dark red to bright yellow) highlighting symmetric or asymmetric directivity of regular (c) and topological (g) lattices. d,h) Snapshots of experimentally acquired wave fields in regular (d) and topological (h) lattices, confirming symmetric or asymmetric propagation. Colors denotes the magnitude of the scan point velocities, normalized by the largest value at the considered instants. a–h) Reproduced with permission.^[^ ^40^ ^]^ Copyright 2018, American Physical Society.

For example, in our previously studied regular (**Figure** **2**a) and topological (Figure 2e) Kagome lattices,^[^ ^40^ ^]^ we calculated the 2D eigenfrequency (Figure 2b,f) and group velocity (Figure 2c,g) contours. Indeed, when exciting the lattice with a point excitation and a burst wave packet, we experimentally observed symmetric and asymmetric wave propagation in the regular (Figure 2d) and topological (Figure 2h) Kagome lattices, in conformance with their corresponding group velocity contours of Figure 2c,g, respectively. Such directionality analysis helps understand the wave patterns due to unit cell symmetry at each frequency. For example, the group velocity profile can inform us of the deformation patterns in a cellular structure when suffering from an impact. One can also control wave directionality by manipulating unit cell configurations.^[^ ^41^, ^42^ ^]^ Despite these important potentials, the analysis and applications of wave propagation directionality via group velocities are quite sporadic in micro‐ and nanoscale PnCs and AMMs. On the other hand, group velocity calculations have been quite commonly performed to predict the thermal conductivity of nanomaterials in general in many theoretical studies.

## 3 Size Effect on Phononic Crystals and Acoustic Metamaterials

In PnCs and AMMs, both host material properties and unit cell sizes dictate their phononic dispersion relations and phonon wavelengths. Generally speaking, bandgap frequencies are inversely proportional to the characteristic length of a unit cell. For microstructures, waves can be manipulated at frequencies in the MHz–GHz regime (ultrasound to hypersound) with long traveling distances, whereas that in nanostructures are in THz (heat transfer) with shorter wavelengths, where phonons can also easily couple with electrons and photons in the same frequency range.^[^ ^27^, ^43^ ^]^ Thus, although the same principles alluded to in Section 2.1 should apply to PnCs and MMs at both length scales, different approaches need to be taken in both theoretical predictions and experimental realization and characterization.

Despite the difference in wavelengths in the applications of these two scales, the term PnC or AMM should be chosen according to the unit cell size compared with the wavelength of interest. For example, at the microscale, decorated membrane resonators separated by deep subwavelength (by two orders of magnitude smaller than the relevant wavelength in the air) have been used as an AMM to absorb low‐frequency sound waves.^[^ ^44^ ^]^ On the other hand, a hexagonal array of snowflake‐shaped inclusions with the unit cell size comparable with the wavelength etched into a thin plate opening a bandgap in GHz is considered a PnC.^[^ ^45^ ^]^ A similar nomenclature convention applies to nanomaterials. Nonetheless, at the nanoscale, phonon transport beyond sub‐Kelvin (below 10 K) temperatures is highly nonlinear and a broader frequency spectrum of phonons contributes to the overall thermal conductivity. More phonon characteristics, including phonon group velocities, phonon scattering, and phonon lifetime, in addition to the phonon dispersion relation, determine phonon transport. Further discussions on nanoscale PnCs and MMs are presented in Section 3.2.

### 3.1 Theoretical Analysis for Microscale Phononic Crystals and Acoustic Metamaterials

As PnCs and AMMs scale down to the microscale, fabrication and assembly processes with high‐precision pose more challenges due to the small scale. Although tremendous progress has been made in micro‐ and nanofabrication, special care still needs to be taken to ensure the strength of constituents, the accuracy in details, and the uniformity of all the repeated cells. Several review articles have discussed the recent progress and challenges in micro‐ and nanofabrication of PnCs and AMMs.^[^ ^9^, ^27^, ^46^ ^]^ Hence, high‐accuracy mathematical models making reliable predictions on the physical properties of the PnCs and AMMs are critical to alleviating experimental efforts. In this section, a brief overview of the commonly applied mathematical models for phononic wave propagation in PnCs and AMMs, the methodologies to optimize the topology for maximizing the desirable effects, and the intrinsic nonlinearity in all PnCs and AMMs, but could be more evident at the microscale with host materials being thin films or soft matters, or undergoing large deformation and vibration compared with its own size, are provided.

#### 3.1.1 Computational Methods for Wave Propagation

To predict phonon wave propagation for realistic microscopic PnCs and AMMs, the simple mass‐spring systems presented in Figure 1 are usually insufficient to account for system complexity. In linear elastic systems at the microscale, one often resorts to continuum mechanical models like those used in macroscale systems. One of the most common approaches is to conduct the unit cell analysis using direct numerical FEA with Bloch–Floquet boundary conditions for the phonon dispersion relations and eigenmodes (mode shapes),^[^ ^39^, ^47^, ^48^, ^49^, ^50^ ^]^ which is straightforward to implement and also readily available in commercial software, such as COMSOL Multiphysics. However, to achieve high accuracy, dense meshes with sufficiently small mesh sizes of systems are needed, making it computationally expensive. Other computational methods, such as the transfer matrix method,^[^ ^51^, ^52^, ^53^, ^54^ ^]^ the generalized differential quadrature method,^[^ ^53^ ^]^ the generalized homogenization framework,^[^ ^55^ ^]^ the planewave expansion method,^[^ ^56^, ^57^, ^58^, ^59^, ^60^, ^61^ ^]^ the reduced Bloch‐mode expansion method,^[^ ^62^ ^]^ the finite‐difference time domain method,^[^ ^56^, ^63^, ^64^ ^]^ the multiple‐scattering theory,^[^ ^16^, ^65^, ^66^, ^67^, ^68^ ^]^ perturbation theory,^[^ ^69^ ^]^ and the spectral variational multiscale model,^[^ ^70^ ^]^ although with different levels of accuracy and efficiency, can more often produce phonon dispersion relations with higher fidelity and/or computational efficiency without significant deviation from the realistic systems compared with direct numerical FEA. Recently, a semianalytical solution of 1D boundary value problems governed by the wave equation in periodic media at arbitrary frequencies has been proposed,^[^ ^71^ ^]^ which is also highly effective and allows for tunability of wave propagation in periodic materials by adjusting boundary conditions only. A generalized Bloch's theorem has also been proposed to solve for viscous 1D PnCs and AMMs with multiple attenuation mechanisms, including spatial attenuation due to Bragg scattering and temporal energy loss.^[^ ^72^ ^]^

Despite the improved efficiency and accuracy of nondirect numerical FEA, their applications are mostly at the macroscale. It is because, on the one hand, studies of PnCs and AMMs at the microscale often focus only on a qualitative agreement between the theoretical design and experimental measurement due to experimental challenges. On the other hand, these nondirect numerical FEA methods typically require either a simple configuration of PnCs and AMMs or lumping several components or materials into one piece using their effective properties, which may oversimplify the problem and cause ignorance of critical details, especially the intricate interactions at the interface between different materials or geometries. In addition, many of these analyses are only suitable for low‐dimensional structural dynamics. Increased dimensionality and architectural details will lead to enhanced complications of mathematical models. Hence selecting the appropriate numerical method according to the expected wave properties, geometric complexity, and the availability of computational resources is crucial in predicting the wave behaviors effectively.

Once phonon dispersion relations are obtained to understand phononic wave properties in bulk, full‐scale dynamic simulations are often performed to confirm the temporal response of bulk wave propagation.^[^ ^50^ ^]^ To acquire such a response, time integration of the general form of a dynamic finite element is performed, which can be written as

(10) $$M\ddot{u}+C\dot{u}+Ku=F$$

where ***M***, ***C***, and ***K*** are mass, damping, and stiffness matrices, respectively, and ***F*** is the force vector. In structural dynamics, one of the most widely used numerical methods is the Newmark‐*β* method^[^ ^73^ ^]^ due to its flexibility in application.^[^ ^74^ ^]^ The n+1th time step displacement $u_{n+1}$ and velocity ${\dot{u}}_{n+1}$ can be obtained via the displacement, velocity, and acceleration information at time step *n* by solving the following three equations.

(11) $$M{\ddot{u}}_{n+1}+C{\dot{u}}_{n+1}+Ku_{n+1}=F_{n+1}$$

(12) $$u_{n+1}=u_{n}+\Delta t{\dot{u}}_{n}+\frac{\Delta t^{2}}{2}[(1-2\beta ){\ddot{u}}_{n}+2\beta {\ddot{u}}_{n+1}]$$

(13) $${\dot{u}}_{n+1}={\dot{u}}_{n}+(1-\gamma )\Delta t{\ddot{u}}_{n}+\gamma \Delta t{\ddot{u}}_{n+1}$$

where *γ* and *β* are user‐defined variables with 0≤γ≤1 and $0\leq \beta \leq \frac{1}{2}$. One of the common combination of these two variables is $\gamma =\frac{1}{2}$ and $\beta =\frac{1}{4}$, which follows the middle point rule and is unconditionally stable. A combination of $\gamma =\frac{1}{2}$ and β=0, that is, the velocity Verlet algorithm,^[^ ^75^ ^]^ is stable only when ωΔt≤2 and is usually used in molecular dynamics (MD) simulation, which is a common simulation method used in phononic studies of nanoscale PnCs and AMMs.

Despite the advantages of its flexibility, the Newmark‐*β* method suffers from several disadvantages. For example, the Newmark‐*β* method can only achieve a second‐order accuracy when $\gamma =\frac{1}{2}$; for all other *γ* values, the method is only first‐order accurate. When $\gamma =\frac{1}{2}$, it lacks the high‐frequency algorithm damping, thus yielding spurious results at high frequencies for systems with high degrees of freedom. Hence, other methods have also been proposed to analyze the dynamics in PnCs and AMMs. Runge‐Kutta is one of the examples that have been applied to study the gaps in amplitude for elastic soliton AMMs.^[^ ^76^ ^]^ Although noted for its high accuracy, stability, convergence, and generality in its application, Runge–Kutta is not always desirable due to its relatively low computational efficiency.^[^ ^74^ ^]^ Later in 1993, a generalized‐*α* method was proposed by Chung and Hulbert^[^ ^77^ ^]^ and is more accurate, stable, and generates proper dissipation at high frequency. However, Erlicher et al.^[^ ^78^ ^]^ proved the overshoot behavior of this method in nonlinear dynamics. More recently, the overshoot issue was resolved by lowering the order of the second‐order governing equation, Equation (10) by expressing $\ddot{u}$ as $\dot{v}$ where ***v*** is the velocity vector.^[^ ^79^, ^80^ ^]^ In addition, many other time‐integration algorithms have been developed, some of the most prominent ones include the Wilson‐*θ* method,^[^ ^81^ ^]^ the HHT‐*α* method^[^ ^82^ ^]^ (implemented in ANSYS and ABAQUS), the G‐*α* method,^[^ ^83^ ^]^ the WBZ‐*α* method,^[^ ^84^ ^]^ the HP‐$\theta_{1}$ method,^[^ ^85^ ^]^ and the collocation method.^[^ ^86^ ^]^ When selecting the time‐integration algorithm, there is always a trade‐off between computational accuracy and efficiency. Hence, one should make decisions based on their priorities.

#### 3.1.2 Topology Optimization Methods

Another vital contribution to the theoretical analysis of microscopic (and macroscopic) PnCs and AMMs is topology optimization, which originated from structural engineering in 1988^[^ ^87^ ^]^ and is aimed at mathematically searching for the best topology within a given design space to maximize expected wave performance. In PnCs and AMMs, topology optimization methodologies have been developed to treat continuous geometries as discrete points and perform selection (and deselection) of them based on target functionality. For example, to find the maximum normalized bandgap between bands *m* and m+1, we can set the objective function as^[^ ^88^, ^89^, ^90^, ^91^, ^92^ ^]^

(14) $$\text{Max}:f(x^{e})=2\frac{{\text{min}}_{q}:\omega_{m+1}(q)-{\text{max}}_{q}:\omega_{m}(q)}{{\text{min}}_{q}:\omega_{m+1}(q)+{\text{max}}_{q}:\omega_{m}(q)}$$

where min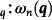 and max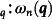 denote the minimum and maximum of the *m*‐th eigenfrequencies $\omega_{m}$ over the entire reciprocal space for a given design of the unit cell $x^{e}$. To find the maximum value of the objective function, we can analyze its sensitivity. Take an undamped system with a governing equation similar to Equation (4) but with a higher dimensionality (and thus wavevector ***q***, instead of the wave number, *q*, in the equation) as an example, the sensitivity of eigenfrequency at wavevector ***q***, ω(q) with respect to the design variable $x^{e}$ (i.e., structural or material properties), can be acquired from^[^ ^93^ ^]^

(15) $$\frac{\partial \omega (q)}{\partial x^{e}}={\tilde{u}}^{T}\frac{\partial (K-\omega^{2}(q)M)}{\partial x^{e}}\tilde{u}$$

**Figure 3:**
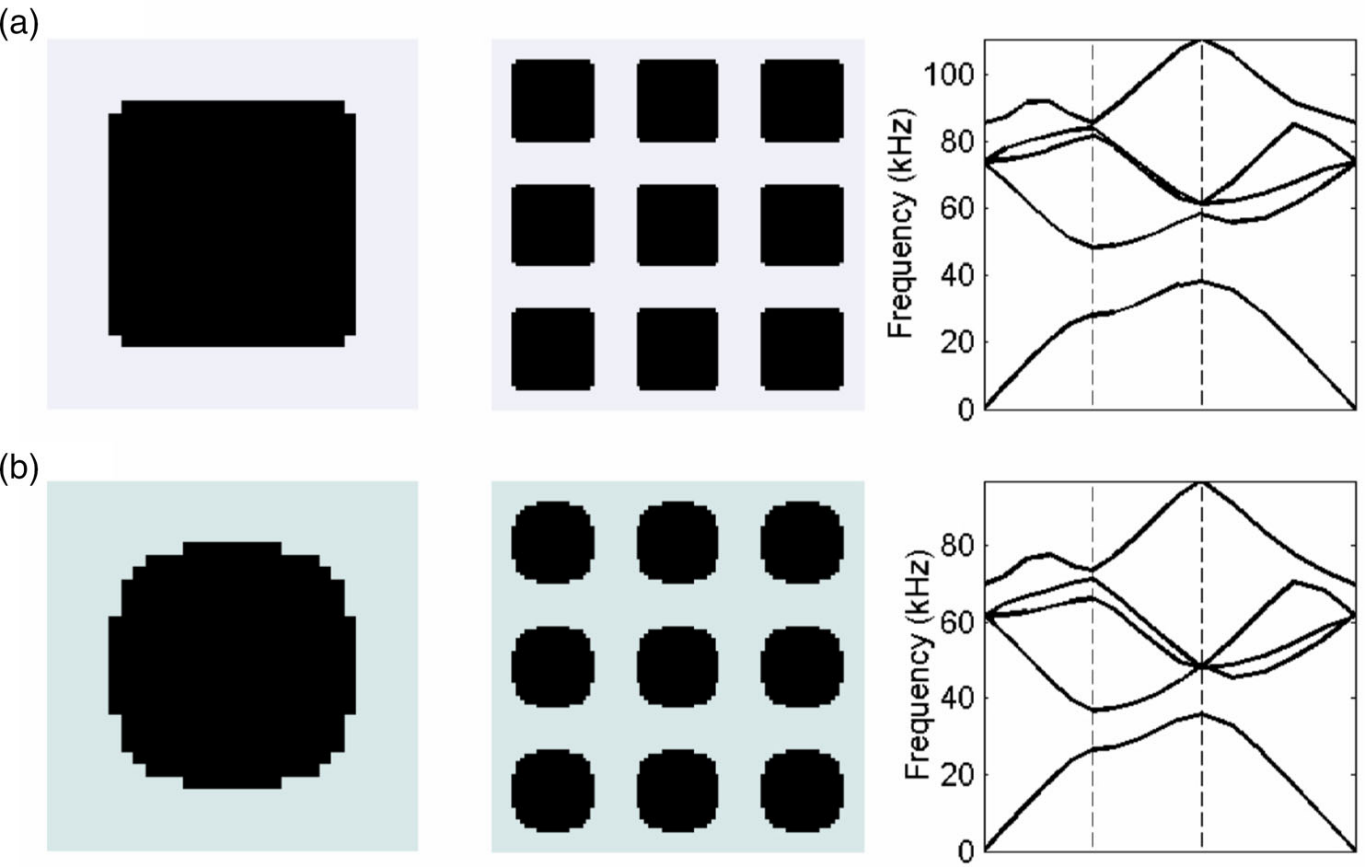
a,b) Examples of material optimization via a GTO method using a high‐contrast case (a) and a low‐contrast case (b) to achieve desired bandgaps. a,b) Reproduced with permission.^[^ ^107^ ^]^ Copyright 2003, The Royal Society Publishing.

Such an optimization via a gradient of the property is called the gradient‐based topology optimization (GTO) method. Several GTO methods have been developed to study periodic structures/materials, such as solid isotropic material with the penalization method,^[^ ^94^, ^95^ ^]^ bidirectional evolutionary structure optimization (BESO) method,^[^ ^96^, ^97^, ^98^, ^99^, ^100^ ^]^ and the level set method,^[^ ^101^, ^102^ ^]^ to obtain many desirable mechanical quantities, such as high stiffness,^[^ ^103^ ^]^ optimal phonon transmission coefficient,^[^ ^104^ ^]^ switchable phonon wave manipulation,^[^ ^105^ ^]^ topologically robust edge states,^[^ ^106^ ^]^ and bandgap optimization of PnCs and AMMs,^[^ ^88^, ^107^, ^108^ ^]^ as shown in **Figure** **3**.

However, as shown in Equation (15), the GTO method relies heavily on the initial design and can easily get trapped by local optima, thus facing complications when multiple localized solutions exist. It can also be challenging to conduct a sensitivity analysis when the gradient information is difficult to obtain, particularly in multiphysics problems with multiple optimization targets. On the other hand, non‐GTO (NGTO) methods, such as the genetic algorithm (GA),^[^ ^109^ ^]^ can avoid these issues by searching for a certain objective function among a wide pool of candidate solutions. This step is called selection. A group of operators will then be applied to evolve the solution to a more suitable design with higher objective function values via crossover to hybridize the selected materials/structures (typically referred to as chromosomes) that survive selection and mutation to randomly alter characteristics of the optimized chromosomes to prevent premature convergence to a suboptimal result.^[^ ^110^ ^]^ Such a method was inspired by biological evolution mechanisms in nature and has been more widely applied to optimize the performance of PnCs and AMMs, such as unit cell design for bandgap optimization^[^ ^110^, ^111^, ^112^, ^113^, ^114^, ^115^ ^]^ and optimizing sonic crystal attenuation properties by creating vacancies,^[^ ^116^ ^]^ due to their resistance to local optima, a lack of reliance on derivatives, the ability to optimize functions of continuous and discrete parameters, and the feasibility to be implemented in parallel.^[^ ^110^ ^]^ However, it is worth noting that despite the more exhaustive design space, NGTO provides for topology optimization compared with GTO, implementing that NGTO typically requires sufficient computational resources. One needs to determine which method to apply based on whether such a comprehensive search is necessary, depending on the complexity and self‐adjointness of the optimization problems.^[^ ^117^ ^]^ More detailed reviews on topology optimization of PnCs and AMMs can be found in other studies.^[^ ^118^, ^119^ ^]^

In addition to the aforementioned GTO and NGTO methods, the advent of machine learning (ML) techniques has provided more convenient pathways for material/structural topology optimization. For example, Liu et al.^[^ ^120^ ^]^ implemented a generative, deep, network model to reversely design and optimize the unit cells of photonic metasurfaces to respond to user‐defined, on‐demand input spectra. Long et al.^[^ ^121^ ^]^ trained the network for forward prediction and inverse design based on the pretrained tandem pipeline to achieve the optimal structure for target topological properties. Liu et al.^[^ ^122^ ^]^ applied both supervised and unsupervised neuron network (NN) models to realize the intelligent inverse design of 1D PnCs and demonstrated the superiority of NN methods compared with GA. These three examples were among some of the earlier studies on the design of photonic and PnCs and MMs via ML. Subsequently, a flux of ML algorithms have been developed to achieve an intelligent on‐demand design of PnCs and AMMs with details listed in a recent review.^[^ ^123^ ^]^

**Figure 4:**
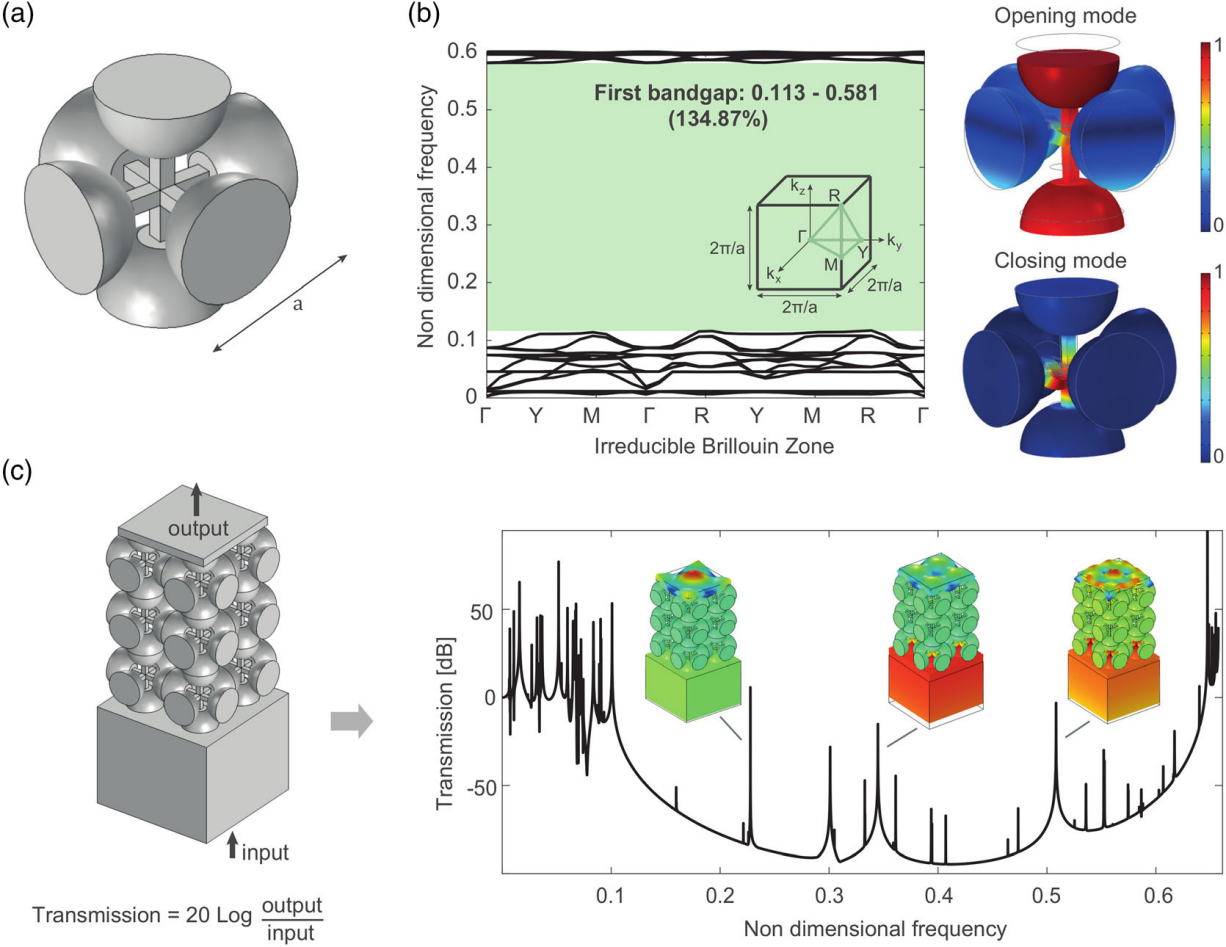
a) A unit cell of the PnC inspired by the optimized topology with a wide bandgap in the study by D’Alessandro et al.^[^ ^99^ ^]^ b) Phonon dispersion relation along the irreducible Brillouin zone presenting a wide bandgap with opening and closing modes. c) Nondimensional transmission diagram computed on a 2 × 2 × 3 PnC. a–d) Reproduced under the terms of the CC‐BY Creative Commons Attribution 4.0 International license (https://creativecommons.org/licenses/by/4.0).^[^ ^128^ ^]^ Copyright 2022, The Authors, published by MDPI.

Our above‐listed example applications of topology optimization in PnCs and AMMs are all implemented at the macroscale. Although such an effort can be easily transitioned to the microscale, as has been done in photonic crystals and MMs,^[^ ^124^, ^125^, ^126^, ^127^ ^]^ curiously, it has been only sporadically implemented in PnCs and AMMs at the same length scale. One of the closest examples is realized by Zega et al.^[^ ^128^ ^]^ who designed 3D PnCs at the microscale with a large bandgap with a gap to the midgap ratio of 134.87% and confirmed its existence with experiment, as shown in **Figure** **4**. Nonetheless, this study was not a direct result of topology optimization; instead, the configuration was inspired by the topologically optimized 3D PnCs composed of a single material endowed with an ultrawide complete bandgap investigated by D’Alessandro et al.^[^ ^99^ ^]^ Hence, the design of PnCs and AMMs at the microscale will benefit tremendously from the various proposed topology optimization methods to achieve desired properties, which have been demonstrated to be helpful at the macroscale PnCs and AMMs, as well as microscale photonic crystals.

#### 3.1.3 Nonlinear Effect

On the one hand, at the microscale, a small mechanical deformation due to either vibration or static forces may easily exceed the linear mechanics regime due to the structure's small size, resulting in nonlinear behaviors that may yield various intriguing phenomena. When deformation is caused by blasts or ballistic impacts, severe nonlinearity and complex inelastic deformation may also occur.^[^ ^46^ ^]^ On the other hand, a growing effort has been devoted to obtaining the structural control of microscale phononic/photonic crystals and MMs by synthesizing reconfigurable MMs using soft matter due to their flexibility^[^ ^129^, ^130^, ^131^, ^132^, ^133^ ^]^ endowed with intrinsic material nonlinearity. Unlike in hard materials under prestress, incorporating both material and geometric nonlinearity in the analysis of dynamics is crucial in understanding the phononic wave propagation in soft micro‐PnCs and AMMs due to their compliance to large deformations. Despite the importance and ubiquity of nonlinearity in microscale PnCs and AMMs, a deep understanding of their nonlinear behaviors and applications is still lacking. In this section, some common nonlinearities that have been intensively investigated in periodic materials are summarized. These theories can potentially inspire the future study of nonlinear wave propagation in microscale PnCs and AMMs.

The nonlinear analysis in soft micro‐PnCs and AMMs falls within the general principles of nonlinear continuum mechanics. Consider a point with position vector ***X*** in the undeformed body. After the material undergoes deformation, the particle originally located at ***X*** moves to a new location ***x*** after a time period of *t* via an operation ***χ***.

(16) x=X(X,t)

The spatial gradient of deformation is defined as

(17) $$F=\frac{\partial \chi }{\partial X}$$

In the absence of body force, divergence of the first Piola–Kirchhoff stress tensor, $S_{0}$, reduces to zero.

(18) $$\text{Div}S_{0}=\rho_{0}\frac{D^{2}\chi }{Dt^{2}}=0$$

where $\rho_{0}$ is the density in the reference configuration, D/Dt denotes the material total time derivative, and $S_{0}$ is defined as

(19) $$S_{0}=JF^{-1}\sigma_{0}$$

where $\sigma_{0}$ is the Cauchy stress tensor and *J*=det ***F***. Subscript “0" indicates the variables with respect to the predeformed configurations.

For a compressible hyperelastic constituent material, $S_{0}$ can be calculated from the strain energy density function *W*.

(20) $$S_{0}=\frac{\partial W}{\partial F}$$

where *W* can be described by various hyperelastic models, such as Neo–Hookean,^[^ ^134^, ^135^ ^]^ Gent,^[^ ^136^ ^]^ Arruda–Boyce,^[^ ^137^ ^]^ Buche–Silberstein,^[^ ^138^ ^]^ Mooney–Rivilin,^[^ ^139^, ^140^ ^]^ Fung,^[^ ^141^ ^]^ Yeoh,^[^ ^142^ ^]^ and Ogden^[^ ^143^ ^]^ models, that describe the nonlinear stress–strain relationships.

Consider a time‐variant infinitesimally incremental perturbation in displacement $u(X,t)=\dot{x}(X,t)$ superimposed on finite predeformation, where the superposed dot denotes a small increment in the physical quantity. The incremental wave motion equation in reference to the undeformed configuration then becomes

(21) $$\text{Div}{\dot{S}}_{0}=\rho_{0}\frac{D^{2}u}{Dt^{2}}$$

and ${\dot{S}}_{0}$ is

(22) $${\dot{S}}_{0}=A_{0}\dot{F},{\dot{S}}_{0ij}=A_{0ijkl}{\dot{F}}_{kl}$$

where

(23) $$\dot{F}=\frac{\partial u}{\partial X},{\dot{F}}_{ij}=u_{i,j}$$

and ***A*** is a fourth‐order incremental elastic tensor whose components are given by

(24) $$A_{0ijkl}=\frac{\partial^{2}W}{\partial F_{ij}\partial F_{kl}}$$

The instantaneous forms of density (*ρ*), the small increment of elastic tensor (***A***), and the first Piola–Kirchhoff stress tensor ($\dot{S}$) along with its divergence can be expressed as

(25) $$\rho =J^{-1}\rho_{0}$$

(26) $$A_{ijkl}=J^{-1}F_{im}F_{kn}A_{0mjnl}$$

(27) $$\dot{S}=A\dot{F}$$

(28) $$\text{Div}\dot{S}=\rho \frac{D^{2}u}{Dt^{2}}$$

Substituting Equation (25) and (27) into Equation (28), we get

(29) $$A_{ijkl}u_{k,lj}=\rho \frac{D^{2}u_{i}}{Dt^{2}}$$

Rewriting Equation (29) using a planewave assumption with Bloch boundary conditions, that is, $u(X,t)=u_{0}e^{i(\omega t-qX)}$, then yields

(30) $$(A_{ijkl}q_{j}q_{l}-\rho \omega^{2})u_{0}=o$$

with ***q*** being the wavevector in the reciprocal space and *ω* the eigenfrequencies (consistent with our prior definition). The eigensolutions of Equation (30) rely heavily on ***A***, which depend on the hyperelastic strain energy density function, *W*. Such a relationship can help predict the phonon wave properties, including the dispersion relations, at different levels of deformation.

As shown, selecting the right *W* closest to reality is crucial in accurately predicting phonon wave propagation. A comparison of several nonlinear models can be found in other studies.^[^ ^144^, ^145^, ^146^, ^147^ ^]^ Although remarkable progress has been made in nonlinear behavior modeling, more effective and accurate methodologies still remain to be proposed to facilitate the analysis of soft matter mechanics.^[^ ^148^ ^]^ A good understanding of the polymer (and/or other material constituents) dynamics at the nanoscale is the key to a realistic description of the stress–strain relationship.

The above material and geometric nonlinear framework are often used along FEA to analyze the wave propagation in PnCs and AMMs composed of soft materials. Various wavelengths of buckling patterns can occur due to different levels of instability. For buckling wavelength much longer than the microstructure size, the ellipticity of the homogenized continuum is lost and is referred to as macroscopic instability. Otherwise, microscopic instability takes place, where the Bloch–Floquet analysis can be applied to examine such instability.^[^ ^149^, ^150^, ^151^ ^]^ In PnCs and AMMs, phonon waves can be tuned by inducing different instabilities.^[^ ^149^, ^152^, ^153^, ^154^ ^]^ Nevertheless, despite its applicability at the microscale, especially when the constituent materials are hyperelastic thin films, only a few studies^[^ ^155^, ^156^ ^]^ have taken advantage of a combination of material and geometric nonlinearity to tune their phononic wave propagation.

In addition, harmonic tuning mechanisms have also been realized by including nonlinearity in PnCs and AMMs, such as subharmonic generation, where a fraction of the propagating energy is transferred to frequencies that are fractions of the exciting frequency, preventing wave propagation when generated subharmonic frequencies are located in a bandgap,^[^ ^157^, ^158^ ^]^ or vice versa,^[^ ^159^ ^]^ and zero‐frequency generation, which is due to wave self‐interactions with asymmetric nonlinearity that causes rectification of an excited wave, leading to unit cell displacements from their original equilibrium position upon wave propagation through them^[^ ^160^, ^161^ ^]^ and the expansion of phononic systems.^[^ ^161^, ^162^, ^163^ ^]^

**Figure 5:**
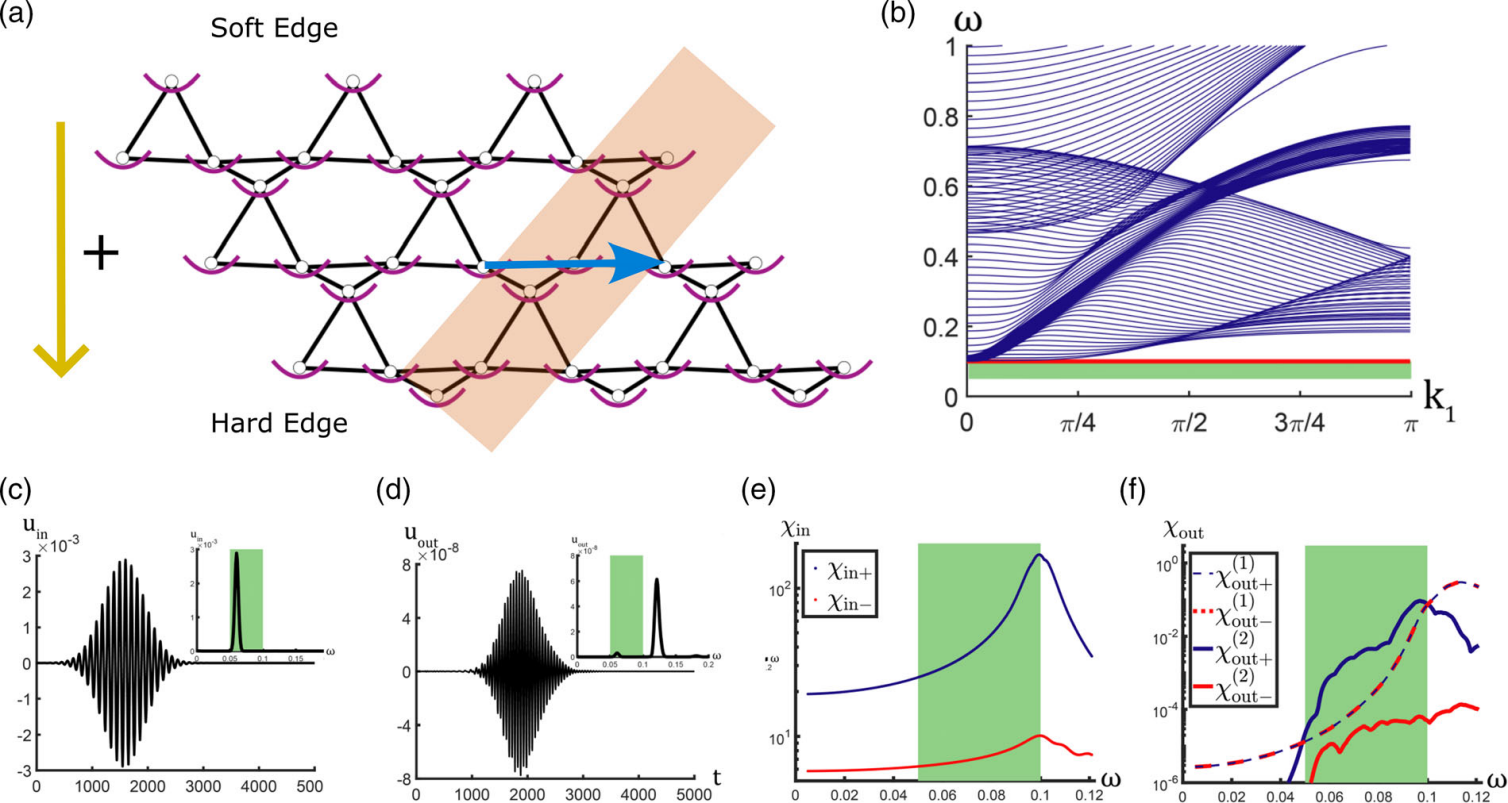
a) Schematic illustration of the topological Maxwell lattice with on‐site potential. The shaded region is a strip of the supercell that can be replicated along the blue arrow direction (i.e., 1D lattice vector). The lattice polarization vector (not shown) points to the top edge, where we denote it as the soft edge, while the hard edge is located at the bottom of the lattice. Transmission from the soft (hard) to the hard (soft) edges is denoted as the positive (negative) direction. b) Phonon dispersion of supercell strip shaded in (a) with on‐site potential. The onset of the edge mode excitation frequency is marked with a red line. The frequency range where second harmonics are in the band is marked with a green strip. c,d) Time history of input‐end (c) and output‐end (d) displacements for monochromatic point excitation in the form of Gaussian tone burst (frequency spectra in the inset). e) Input‐end and f) output‐end frequency response (i.e., transmission, denoted as *χ*) for tone burst excitation. Blue (red) curves denote the transmissions measured at the hard (soft) edge. Subscript “in” (“out”) denotes the frequency response at the input (output) location. The solid (dashed) curves in (f) represent the second (first)‐harmonic responses. a–f) Reproduced with permission.^[^ ^187^ ^]^ Copyright 2020, American Physical Society.

Among all the harmonic tuning mechanisms, the generation of higher harmonics via nonlinearity^[^ ^164^ ^]^ has been investigated to a greater extent in the phononics community. By generating higher harmonics in periodic systems, a fraction of the energy is transferred to frequencies that are multiples of the excitation frequency.^[^ ^161^, ^163^, ^165^, ^166^, ^167^, ^168^, ^169^, ^170^, ^171^, ^172^, ^173^, ^174^, ^175^, ^176^, ^177^, ^178^, ^179^, ^180^, ^181^, ^182^, ^183^, ^184^, ^185^, ^186^ ^]^ Such an energy transfer enables many important properties, such as modal interaction,^[^ ^165^, ^171^, ^179^ ^]^ intermodal hopping,^[^ ^170^ ^]^ self‐switching functionality selection capabilities,^[^ ^169^ ^]^ bandgap tunability,^[^ ^174^ ^]^ and wave reflection manipulation.^[^ ^175^, ^176^ ^]^ Recently, some of the efforts have been devoted to breaking the reciprocity to create phonon diodes by generating higher harmonics.^[^ ^187^, ^188^ ^]^ For example, by leveraging a combination of asymmetry associated with the topologically protected floppy edge modes in a topological Maxwell lattice and geometric nonlinearity built into the mechanical system responses, we were able to achieve a nonreciprocal wave propagation of the second harmonic response of the initial excitation. With additional on‐site pinning potentials (which lift the topological floppy edge mode frequency to $\omega_{0}$) that block the linear transmission, a phonon diode allowing only one‐way signal transmission was created,^[^ ^187^ ^]^ as presented in **Figure** **5**. When exciting the lattice within a frequency range $\omega_{0}/2<\omega <\omega_{0}$ (within the green strip in Figure 5b), the larger deformation at the soft edge compared with that at the hard edge triggers a stronger second‐harmonic wave propagation at 2*ω* residing in the bulk band, thus presenting an asymmetric nonlinear response. Although the topological Maxwell lattice topology is asymmetric, nonreciprocity cannot be achieved without nonlinearity. From Figure 5f, we can see that the second‐harmonic waves excited from the soft edge can even present a higher amplitude compared with that of the first harmonic (i.e., the linear response), the latter of which presents a reciprocal behavior in phonon transmission when exciting from two different edges.

Other nonlinear properties, such as wave mixing,^[^ ^189^, ^190^ ^]^ supratransmission,^[^ ^191^, ^192^, ^193^, ^194^, ^195^, ^196^ ^]^ and solitary waves,^[^ ^197^, ^198^, ^199^, ^200^, ^201^ ^]^ that have been extensively investigated in PnCs and AMMs at the macroscale, may also be potentially applicable at the microscale with minor adjustments. A more comprehensive review on the nonlinearity of phonon wave propagation in PnCs and AMMs can be found in the study by Patil et al.^[^ ^202^ ^]^

#### 3.1.4 Phonon Manipulation in Microscale Phononic Crystals and Metamaterials

**Figure 6:**
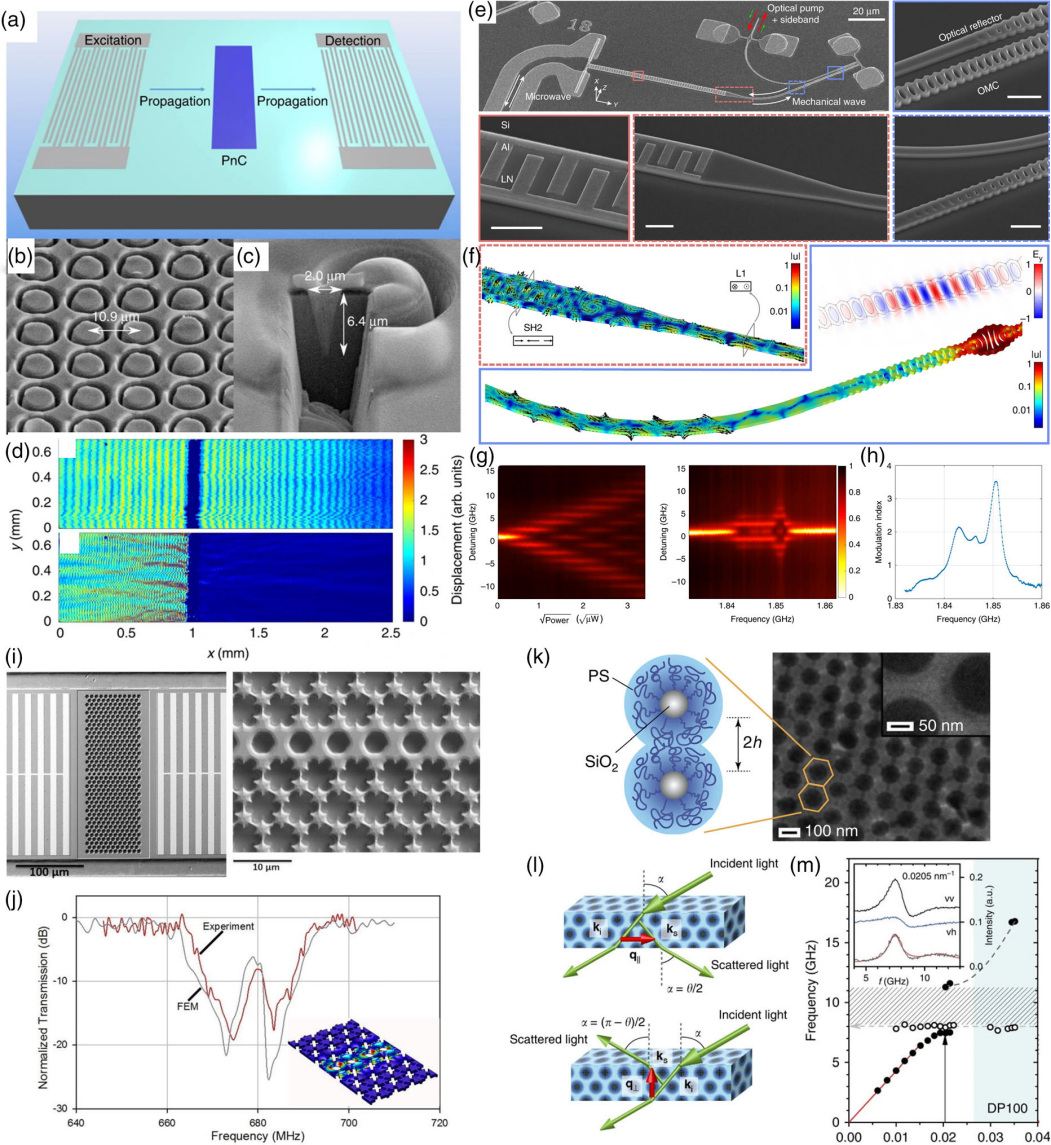
a) Schematic of device used to characterize the PnC shown in (b), with details of a unit cell in (c). d) Measured out‐of‐plane SAW displacement at the propagating and bandgap frequencies. a–d) Reproduced under the terms of the CC‐BY Creative Commons Attribution 4.0 International license (https://creativecommons.org/licenses/by/4.0).^[^ ^203^ ^]^ Copyright 2017, The Authors, published by Springer Nature. e) Scanning electron microscopy (SEM) images of one piezo‐optomechanical transducer with a zoomed‐in image showing the conversion region between microwave and mechanics with a red frame and between mechanics and optics shown in a blue frame. f) FEM simulation of the IDT‐to‐waveguide taper. g) Experimentally measured reflection spectrum of the optical cavity for different microwave power (left) and frequency (right). h) Modulation index for different microwave drive frequencies extracted from the right figure in (g). e–h) Reproduced under the terms of the CC‐BY Creative Commons Attribution 4.0 International license (https://creativecommons.org/licenses/by/4.0).^[^ ^330^ ^]^ Copyright 2020, The Authors, published by Springer Nature. i) SEM image of a fabricated SiC microwaveguide PnC with drive and sense with a zoomed‐in image of the waveguide on the right. j) Experimental and numerical results of the SiC microwaveguide. i,j) Reproduced with permission.^[^ ^45^ ^]^ Copyright 2018, AIP Publishing LLC. k) SEM image of a face‐centered‐cubic packing of the nanoparticles. l) Brillouin light scattering geometries probing phonon propagation along the wavevector $q=k_{s}-k_{i}$, with $k_{s}$ and $k_{i}$ being the incident laser and scattered light wavevectors, respectively. m) Experimental dispersion relation showing a clear bandgap (patterned area) and a localized mode (open circles). k–m) Reproduced under the terms of the CC‐BY Creative Commons Attribution 4.0 International license (https://creativecommons.org/licenses/by/4.0).^[^ ^204^ ^]^ Copyright 2015, The Authors, published by Springer Nature.

Applications of microscale PnCs and AMMs usually resemble those at the macroscale, despite with more difficulties in fabrication and characterization. For example, Ash et al.^[^ ^203^ ^]^ fabricated a surface‐acoustic‐wave (SAW) PnC with finite‐depth annular holes as unit cells to create low‐frequency bandgaps within the sound line and up to an order‐of‐magnitude improved band gap extinction compared with pillared PnCs, shown in **Figure** **6**a–d. By creating a path in the structure inside the bandgap frequency range, Ghasemi Baboly et al.^[^ ^45^ ^]^ created a conventional PnC waveguide in silicon carbide (SiC) to guide waves to transport along the path at frequencies within the bandgap, as shown in Figure 6i,j. Moreover, due to the higher‐phonon frequencies at the microscale, transition between acoustics and optics can be realized at this scale. For example, with a specially fabricated and integrated piezooptomechanical transducer by combining an efficient wavelength‐scale mechanical wave transducer and an optimized optomechanical crystal (OMC) on the lithium niobate on silicon (LNOS) platform, Jiang et al. achieved an on‐chip bidirectional piezo‐optomechanical transduction between microwave and optical frequency, as presented in Figure 6e‐h. The efficiency of such a transducer can be further improved at cryogenic temperature due to reduced material loss for the interdigitated transducer (IDT) and the OMC, opening a new paradigm for integration with quantum sensors and processors. Further scaling down the microscale PnCs and AMMs via the self‐assembly of densely packed polymer‐tethered colloidal particles can form hybridization‐type bandgaps,^[^ ^204^ ^]^ resulting from the avoided crossing of two bands with the same symmetry, which is different from a Bragg bandgap typically opened at either q=π/a or 0. Such hybridization‐type bandgaps are robust against disorders, as shown in Figure 6k–m.

**Figure 7:**
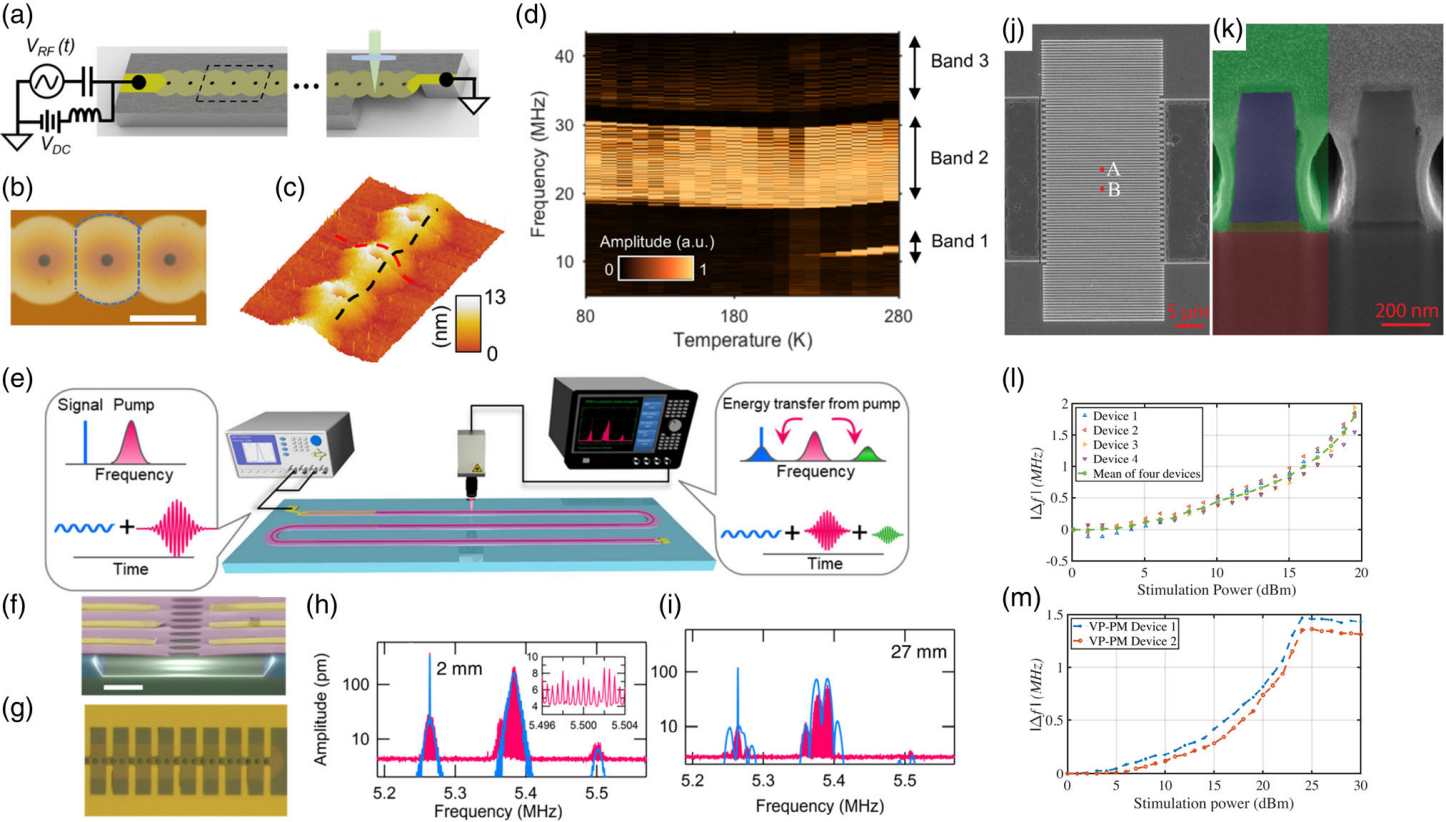
a) Schematic drawing of the phononic waveguide along with the electrostatic actuation and optical detection apparatus. b) Optical image of the waveguide showing inner‐ferometric rings in the silicon nitride membrane due to the out‐of‐plane deflection. The doted line represents a unit cell. The scale bar is 7 μm. c) Isometric topography map of the waveguide acquired from an atomic force microscope showing an out‐of‐plane deflection. d) A 2D map of measured response versus frequency and temperature for the buckled waveguide. a–d) Reproduced with permission.^[^ ^208^ ^]^ Copyright 2021, American Chemical Society. e) A schematic showing the device and the measurement setup for an on‐chip nanoelectromechanical waveguide structure with an IDT. The left and right insets show the input and output signal configurations in a four‐wave mixing experiment. f) A false‐colored SEM image of a cross section with IDT electrodes (yellow). The membrane (pink) is suspended by etching a sacrificial layer (blue). The scale bar is 5 μm. g) A microscopy image of the IDT electrode and the suspended membrane. h,i) Spectral responses of the nanoelectromechanical waveguide measured distances of *x* = 2 mm (h) and 27 mm (i), respectively, when exciting a continuous‐wave signal at 5.264 MHz with a Gaussian‐shaped pump pulse at 5.383 MHz. A pulse idler wave is generated via four‐wave mixing at around 5.502 MHz. e–i) Reproduced with permission.^[^ ^190^ ^]^ Copyright 2020, American Physical Society. j) Top‐view scanning electron microscopy image of the nanostrip phononic metasurface resonator. k) Cross‐sectional view of the high‐aspect‐ratio electrodes. l,m) Power‐dependent frequency shifts of nanostrip (l) and 10‐μm vertically polarized phononic metasurface (m) resonators. Frequency shifts are negative. j–m) Reproduced under the terms of the CC‐BY Creative Commons Attribution 4.0 International license (https://creativecommons.org/licenses/by/4.0).^[^ ^211^ ^]^ Copyright 2021 The Authors, published by AIP Publishing.

A handful of studies also focus on tuning phonon waves by taking advantage of nonlinearity in microscale PnCs and AMMs, as presented in **Figure** **7**. (Please note that here we refer to NEMS cited below as microscale systems since their unit cells and the whole systems are still in micrometers. Systems with unit cells in nanometers are referred to as nanoscale PnCs and AMMs, where phonons are atomic vibrations.) For example, Wallen et al.^[^ ^179^ ^]^ studied the conversion from shear to longitudinal modes via second‐harmonic generation in an imaginary 2D microscale granular crystal. Cha and Daraio^[^ ^205^ ^]^ formed a nonlinear bandgap analogous to the Peierls transition in a NEMS via a dynamic modulation of the voltage and characterized nonlinear second harmonics generated due to the broken out‐of‐plane symmetry of the deflected membranes. Hatanaka et al. observed pulse broadening into higher frequencies in the phonon dispersion relation due to nonlinearity in microscale phononic waveguides.^[^ ^206^ ^]^ Midtvedt et al.^[^ ^207^ ^]^ achieved GHz frequency tuning and coupling by distorting the periodicity of pinned and atomically thin membranes due to geometric nonlinearity. Kim et al.^[^ ^208^ ^]^ used thermally induced buckling due to nonlinearity in nanoelectromechanical phononic waveguides to modulate phonon dispersion relation. Although the membrane itself (made of silicon nitride) is not hyperelastic material in this study, the dispersion‐tuning capability is also a result of a similar mechanical instability mechanism due to material and geometric nonlinearity. As shown in Figure 7d, when the temperature is below 220 K, there is no observable wave transmission, indicating the inactivity of the acoustic band. Moreover, the second passband, although observable throughout the measured temperature range, presents a kink in the direction near the buckling transition around 220 K, where the thermomechanical individual circular plates and beams start to buckle. Once in the postbuckled state, the second‐pass frequency rises due to an increase in the total bending with an additional in‐plane compression.^[^ ^208^ ^]^ Kurosu et al.^[^ ^190^ ^]^ and Hatanaka et al.^[^ ^209^ ^]^ have experimentally demonstrated four‐wave mixing due to mechanical Kerr nonlinearity^[^ ^210^ ^]^ at the microscale, which promises the possibility of generating and fully manipulating a range of nonlinear phenomena using on‐chip mechanical platforms. Specifically, the spectral response of a continuous wave (signal, $f_{s}$) and the intense pulse (pump, $f_{p}$) excited from the IDT generate a new signal (idler, $f_{i}$) due to the four‐wave mixing process, which satisfies the energy‐conservation requirement $2f_{p}=f_{s}+f_{i}$, Figure 7e–i.^[^ ^190^ ^]^ In addition, nanostrip coupling^[^ ^211^ ^]^ may also create nonlinear effects causing a resonance frequency to downshift in a nanostrip phononic metasurface, and the shift increases with the stimulation power due to the enlarged nonlinearity, as shown in Figure 7i. Such nonlinearity also exists in larger‐scale vertical‐polarized phononic metasurfaces, as shown in Figure 7m. However, no theoretical studies has been conducted to confirm this speculation. Hence, we can see the importance of applying appropriate theoretical analysis in deepening the understanding of the inherent complexity and nonlinearity in microscale PnCs and AMMs due to their small scale.

### 3.2 Theoretical Analysis for Nanoscale Phononic Crystals and Metamaterials

Scaling the phononic wave propagation down to the nanoscale requires an atomistic study due to the high vibration frequency (in THz) and extremely short wavelength (in nm and μm) compared with the ones at the microscale (frequencies in MHz–GHz and wavelengths at least in several hundred μm)). At the nanoscale, phonons are due to atomic vibration, which are the main heat carriers in nonmetallic materials (e.g., semiconductors). With the harmonic approximation, phonons travel freely without attenuation, and thus all have an unlimited free path in an infinitely large medium. However, in reality, MFPs are significantly limited (from nm to cm; although the upper bound is in cm, it usually requires an ultra‐low temperature (below 10 K or a sub‐Kelvin temperature), while at the room temperature, the upper bound typically resides in μm) by phonon scattering due to 1) phonon–phonon scattering as a result of the intrinsic nonlinear interatomic interactions at finite temperatures, 2) impurities and defects, and 3) boundaries of the medium. At high temperatures where phonon–phonon scattering is intense, even without any boundaries or defects, phonons cannot travel for a long distance. Therefore, phonon engineering at the nanoscale usually occurs within the ballistic regime where phonons are mainly scattered by boundaries and/or defects, while in the diffusive regime, phonons are less sensitive to such scattering.^[^ ^212^ ^]^

As phonon propagation at the nanoscale is highly sensitive to interatomic interactions which vary constantly at finite temperatures, continuum mechanics methods for phonon propagation analysis used in micro‐ and macroscale PnCs and AMMs as discussed in Section 3.1 often fail to capture all the scattering mechanisms at the nanoscale.^[^ ^213^ ^]^ Researchers usually resort to either classical MD simulations, which are suitable to capture anharmonicity due to the intrinsic nonlinearity of interatomic potentials^[^ ^214^ ^]^ or first‐principle techniques to study phonon transport properties. In this section, fundamentals of thermal conductivity are reviewed and a few popular methods are then listed to obtain a quantitative analysis of phonon transport properties in PnCs and MMs.

In this section, a brief overview of the phonon transport theory and the commonly used theoretical and numerical methods to analyze phonon transport is presented. Then, the various approaches and mechanisms for manipulating phonon transport using nanoscale PnCs and MMs are discussed.

#### 3.2.1 Theory of Phonon Transport

The atomistic theory of thermal conductivity, commonly denoted as *κ*, incorporating various scattering mechanisms treated as perturbations to the harmonic Hamiltonian, was first studied in detail by Klemens,^[^ ^6^, ^215^ ^]^ Carruthers,^[^ ^216^ ^]^ Ziman,^[^ ^217^ ^]^ Callaway,^[^ ^218^ ^]^ and Holland.^[^ ^219^ ^]^ Scattering probabilities are then computed with the Fermi golden rule, while transport properties were captured by the Boltzmann transport equation (BTE) for the probability distribution function $f^{\lambda }$ of the phonons subjected to a thermal gradient^[^ ^220^, ^221^ ^]^ at temperature *T* in the state *λ*.

(31) $$-v_{\lambda }\frac{\partial f_{0}^{\lambda }}{\partial T}\nabla T=-{\frac{\partial f^{\lambda }}{\partial t}|}_{\text{scatt}}$$

where $f_{0}^{\lambda }$ is the equilibrium Bose–Einstein distribution and $v_{\lambda }$ is the particle velocity in the state *λ*. Here the usual assumption of the local equilibrium is made on the left‐hand side of the equation. The solution to this equation can be estimated using the relaxation time approximation (RTA) as a standard approach in first‐principle calculations. Such an approximation is based on: 1) if a probability distribution function for a given phonon mode is not an equilibrium distribution, then the scattering strength is proportional to the deviation from equilibrium with some characteristic time constant *τ* that incorporates all the information about scattering processes, and 2) each relaxation mode is independent of all others, and thus, all other modes are considered to be in equilibrium. With the RTA, Equation (31) can be simplified as

(32) $$-v_{\lambda }\frac{\partial f_{0}^{\lambda }}{\partial T}\nabla T=\frac{f^{\lambda }-f_{0}^{\lambda }}{\tau_{\lambda }}$$

where *τ* is the phonon lifetime that is contributed by all scattering effects, including Umklapp scattering ($\tau_{u}$), mass difference impurity scattering ($\tau_{m}$), dislocation scattering ($\tau_{d}$), boundary scattering ($\tau_{b}$), and is mixed via Matthiessen's rule.^[^ ^222^ ^]^

(33) $$\frac{1}{\tau }=\frac{1}{\tau_{u}}+\frac{1}{\tau_{m}}+\frac{1}{\tau_{d}}+\frac{1}{\tau_{b}}+\ldots$$

Further derivation of Equation (32), including expanding $\frac{\partial f_{0}^{\lambda }}{\partial T}$ and re‐expressing ∇T using *κ* and thermal current, yields the generalized thermal conductivity $\kappa_{\alpha \beta }$.

(34) $$\kappa_{\alpha \beta }=\sum_{\lambda }C_{v}v_{\alpha }^{\lambda }v_{\beta }^{\lambda }\tau_{\lambda },$$

where *α* and *β* are the Cartesian indices, *v* is the particle velocity, and *λ* is denoted as before, the phonon states.

After invoking the Debye approximation for phonon dispersion, *κ* writes as an integral over all possible phonon frequencies *ω*.^[^ ^223^ ^]^

(35) $$\kappa =\frac{v}{3}\int_{0}^{\omega_{d}}C_{v}(\omega )l(\omega )d\omega$$

where *l* is the phonon MFP, that is, the average distance a phonon can travel in a media at *ω*. $\omega_{d}$ denotes the Debye frequency. Heat capacity $C_{v}$ at frequency *ω* takes the form

(36) $$C_{v}(\omega )=3Nk_{B}{\left(\frac{\hbar \omega }{k_{B}T}\right)}^{2}\frac{e^{\frac{\hbar \omega }{k_{B}T}}}{{\left(e^{\frac{\hbar \omega }{k_{B}T}}-1\right)}^{2}}$$

Taking the above integral of Equation (35) yields a simplified form of *κ*

(37) $$\kappa =\frac{1}{3}C_{v}vl,$$

where $C_{v}$ denotes the heat capacity at constant volume, *v* is the average particle velocity, which is the speed of sound at this level of the theory, and *l* is the average phonon MFP, that is, either the characteristic length for the scattering of phonons off one another or off a structural defect, interface, or boundary.

**Figure 8:**
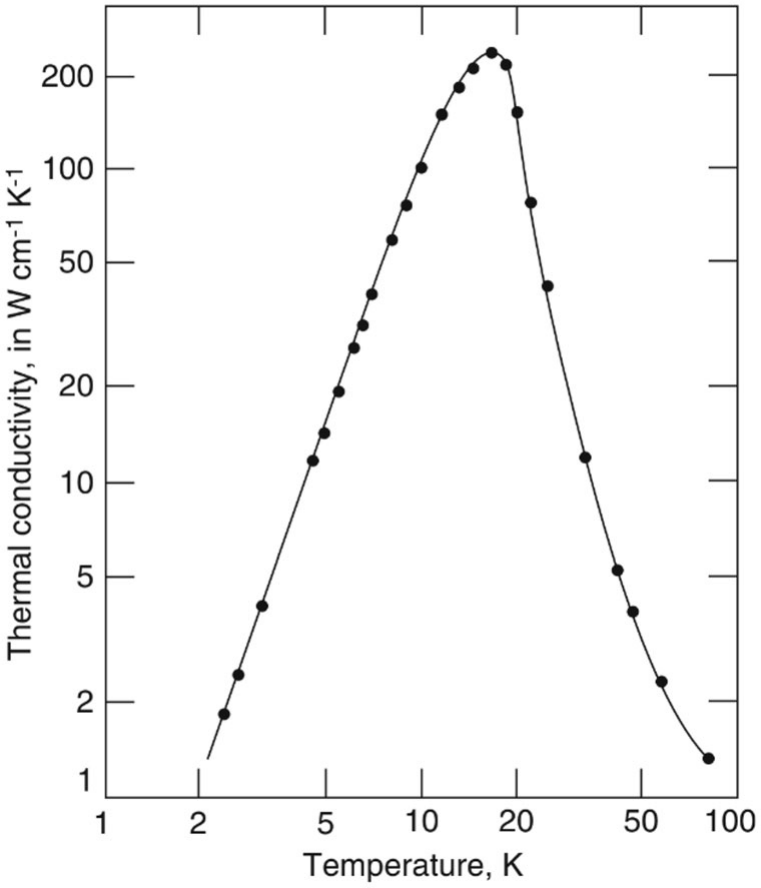
Thermal conductivity of a highly purified crystal of sodium fluoride. At low temperature, $\kappa \propto T^{3}$; at high temperature, $\kappa \propto T^{-1}$. Reproduced with permission.^[^ ^331^ ^]^ Copyright 2021, John Wiley & Sons.

In **Figure** **8**, we see that in the low‐temperature regime, the size effect matters as the coherent phonon transport dominates until phonons come across boundaries or defects, resulting in phonon MFPs being mostly constrained by distances between defects or boundaries. Thus, *κ* can be expressed, instead of Equation (37).

(38) $$\kappa \propto C_{v}vD$$

where *D* is the characteristic length of the specimen, which is related to either the specimen size or averaged interface of impurity distances. At low temperatures, according to the approximation of Equation (36), $C_{v}\propto T^{3}$. Thus $\kappa \propto T^{3}$. Much of the research on engineering thermal transport via the design of PnCs and MMs occurs at low temperatures since phonon transport is highly sensitive to the structural periodicity and is barely affected by Umklapp scattering.^[^ ^213^, ^224^, ^225^, ^226^, ^227^, ^228^, ^229^, ^230^, ^231^, ^232^, ^233^ ^]^

Figure 8 also shows that $\kappa \propto T^{-1}$ at higher temperatures, where, according to Equation (36), $C_{v}$ can be treated as a constant. The total number of excited phonons is proportional to *T*, so that the collision frequency for a given phonon is proportional to the number of excited phonons, resulting in the MFP $l\propto T^{-1}$. Thus, $\kappa \propto T^{-1}$ at higher temperatures. Due to limited phonon MFPs, engineering phonon transport by creating periodic structures presents challenges. Further miniaturization, the use of materials with intrinsically long *l* such as alloys, and improving the fabrication process to eliminate surface roughness can extend the phonon engineering temperatures beyond sub‐Kelvin.^[^ ^234^, ^235^, ^236^, ^237^ ^]^

#### 3.2.2 Molecular Dynamics Simulation of Phonon Transport

Although Equation (37) alluded to in Section 3.2.1 can be used to estimate the *κ* of a material, it usually overestimates *κ* at higher temperatures, where higher‐order scattering processes are neglected in the calculation but contributes to *κ*.^[^ ^238^ ^]^ Moreover, despite high accuracy at relatively low temperatures, first‐principle calculations also suffer from low calculation efficiency for large PnC and MM systems. In this case, many resort to classical MD simulations, which allows for large‐scale simulations without the consumption of many computational resources. Moreover, MD simulation intrinsically includes the description of phonon anharmonicity in interatomic potentials, which is critical in high‐temperature thermal transport in nano‐PnCs and MMs.

In all‐atom MD simulations, each atom is treated as a point mass whose velocity and position are computed via time integration of the classical Newton's equations. The computational task is to solve the set of coupled differential equations given by

(39) $$m_{i}\rho \frac{d^{2}x_{i}}{dt^{2}}=-\frac{\partial \phi }{\partial x_{i}}$$

where $m_{i}$ and $x_{i}$ are the mass and position vector of the *i*‐th particle and $\phi (x_{1},x_{2},\ldots ,x_{n})$ denotes the potential energy ranging from a simple pairwise atomic interaction, such as the Lennard–Jones potential,^[^ ^239^, ^240^ ^]^ to the complicated many‐body potentials, such as Stillinger–Weber three‐body potential,^[^ ^241^ ^]^ and even to the more complicated ab initio fully quantum mechanical methods.

For wave propagation at the macro‐ and microscale PnCs and AMMs, we often start with a unit‐cell analysis by applying Floquet–Bloch boundary conditions to understand bulk wave characteristics. In MD simulations, Bloch boundary conditions, or typically referred to as the PBCs, are applied not only for unit‐cell analysis, but also for investigating full‐scale dynamic simulations to mimic the phonon transport in an infinitely large system while avoiding calculating systems with a large number of atoms. A scaling analysis is usually performed in phonon transport studies to exclude the size effect.^[^ ^242^, ^243^ ^]^

While the interaction of individual atoms is governed by interatomic potentials, as shown in Equation (39), behaviors of the overall system must also follow certain constraints on simulation conditions such as the number of atoms (*N*), the volume (*V*), temperature (*T*), pressure (*P*), and total energy (*E*) of the systems. The most popular combination of constraints, or ensembles, includes the microcanonical (NVE), canonical (NVT), and isothermal–isobaric (NPT) ensembles, with constant quantities represented by letters in the parentheses following these names. When PBCs are applied, choosing the right ensembles for system initialization and phonon transport analysis is vital in obtaining meaningful results. For example, NVT and NPT are usually applied to reach the desired temperature under a constant volume or pressure, respectively. However, despite resembling actual experimental conditions compared with NVE, NVT and NPT unavoidably introduce artificial terms, such as thermostats and barostats, to maintain constant *T* and *P*. On the other hand, transport properties must be analyzed under equilibrium, that is, the exchange of kinetic and potential energies of the system should be under the condition that the total energy (summation of the two) should be conserved, that is, under the NVE ensemble.^[^ ^244^ ^]^

In equilibrium MD, *κ* can be derived from the fluctuation–dissipation theorem and is given by a time integral over the equilibrium flux autocorrelation function, also known as the Green–Kubo method.^[^ ^245^, ^246^ ^]^

(40) $$\kappa_{\alpha \beta }=\frac{V}{k_{B}T^{2}}\int_{0}^{\infty }<j_{\alpha }(0)j_{\alpha }(t)>dt$$

where *V* is the volume of the system. The angular brackets indicate ensemble averages over microstates (*i.e.*, instantaneous heat fluxes) ***j***, which can be expressed as

(41) $$j(t)V=\frac{d}{dt}\sum_{i}x_{i}\varepsilon_{i}$$

where $\varepsilon_{i}$ is the total energy of particle *i*, comprising kinetic and potential components.

(42) $$\varepsilon_{i}=\frac{1}{2}m|v_{i}{|}^{2}+\frac{1}{2}\sum_{j}\phi (r_{ij})$$

Substituting Equation (42) into (41), and considering *ϕ* as a pair‐wise interatomic potential, gives:^[^ ^247^ ^]^

(43) $$j(t)=\frac{1}{V}\sum_{i}\left[v_{i}\varepsilon_{i}+\frac{1}{2}\sum_{j,j\neq i}x_{ij}\cdot (F_{ij}\cdot v_{i})\right]$$

where $F_{ij}$ is the force on atom *i* due to its neighbor *j* from the pair potential *ϕ*. The first term of Equation (43) on the right‐hand side is related to local particle shifts typically occurring in fluids. Thus, in solids, the second term dominates,^[^ ^248^ ^]^ which reduces j to

(44) $$j(t)=\frac{1}{2V}\sum_{i,j,i\neq j}x_{ij}\cdot (F_{ij}\cdot v_{i})$$

For three‐body potentials, such as the Stillinger–Weber potential,^[^ ^241^ ^]^ the three‐body contribution must be taken into account. Therefore, the flux expression is more complicated^[^ ^248^ ^]^

(45) $$j(t)=\frac{1}{V}\left[\sum_{i,j,i\neq j}x_{ij}\times (F_{ij}\times v_{i})+\frac{1}{6}\sum_{i,j,k,i\neq j\neq k}(x_{ij}+x_{ik})\times (F_{ijk}\times v_{i})\right]$$

In MD simulations, the integration in Equation (40) is discretized as

(46) $$\kappa_{\alpha \beta }=\frac{\Delta t}{Vk_{B}T^{2}}\sum_{m=0}^{M}\frac{1}{N-m}\sum_{n=1}^{N-m}j_{\alpha }(m+n)j_{\beta }(n)$$

where *M* is the number of steps over which the ensemble average is calculated, *N* is the total number of time steps once the simulated system reaches equilibrium, and Δt is the MD time step.

It is worth noting that such transport properties are usually calculated under the NVE ensemble. Also, PBCs can be applied to mimic an infinitely large structure. However, a size effect of the periodic box must be investigated to ensure convergence of the calculated *κ*.

In addition to the statistical mechanics‐based Green–Kubo method discussed earlier, we can also use the direct method with Fourier's law for thermal transport under the nonequilibrium MD (NEMD), in which a temperature gradient (e.g., $\frac{\partial T}{\partial z}$, if the temperature gradient is applied in the *z* direction) is imposed on the atomistic sample by thermostatting different “bath” regions at different temperatures. The heat flux *j* along the *z*‐direction can be obtained from the energy exchange rate in the heat baths. *κ* can then be calculated from

(47) $$\kappa =-j{\left(\frac{\partial T}{\partial z}\right)}^{-1}$$

This method is plagued by size effects due to phonon scattering at the thermostated boundaries and restrictions on the maximum phonon MFP. However, we can extrapolate the finite‐size *κ* up to the bulk value with

(48) $$\kappa (L)=\kappa_{\infty }\left(\frac{L_{z}}{L_{z}+4l_{\infty }}\right)$$

where $L_{z}$ is the length of the simulated cell along the *z* direction, for example, and $l_{\infty }$ and $\kappa_{\infty }$ are the phonon MFP and *κ* in an infinite system, that is, bulk crystal or an infinitely long nanowire/nanotube, respectively.^[^ ^242^ ^]^

Care should be taken when using classical MD simulations when the temperature in simulation, $T_{\text{MD}}$, is below the Debye temperature, $T_{D}=\frac{\hbar \omega_{D}}{k_{B}}$. In this case, temperature rescaling is needed to obtain a quantum‐corrected temperature.

(49) $$T_{\text{MD}}=\frac{2T^{3}}{T_{D}^{2}}\int_{0}^{\frac{T_{D}}{T}}\frac{x^{2}}{e^{x}-1}dx$$

In addition, the zero‐point energy should be included in the phonon occupation number when the temperature is above one‐third of $T_{D}$.^[^ ^249^ ^]^

At low temperature, other methods, such as Monte Carlo simulations^[^ ^213^, ^250^, ^251^, ^252^, ^253^, ^254^, ^255^, ^256^ ^]^ and *ab initio* calculations^[^ ^257^, ^258^, ^259^ ^]^ mainly based on the discussion in Section 3.2.1, are more applicable to obtain *κ* and have been largely applied in recent literature on PnCs and MMs. Other methods, such as the Allen–Feldman theory for disordered harmonic solids^[^ ^260^ ^]^ and atomistic Green's function method,^[^ ^261^ ^]^ have also been widely used to study phonon transport, however, with constraints, including the negligence of anharmonicity, which requires further adaptations in their applications.^[^ ^242^, ^262^ ^]^

#### 3.2.3 Other Phonon Interpretation Methods at the Nanoscale

Just like the phonon dispersion relation at the macro‐ and microscale PnCs and AMMs, at the nanoscale, phonon dispersion can also provide important information on the characteristics of phonon transport, such as phonon bandgaps and group velocities. One way to calculate phonon dispersion relations is by analyzing lattice dynamics (LD) under a small displacement approximation, which assumes linear atomic interactions,^[^ ^263^ ^]^ the calculation of which is similar to that presented in Section 2.1.

In theory, group velocity $v_{g}$ can be directly from the phonon dispersion relation, as has been discussed in Section 2.1.2. Alternatively, we can calculate $v_{g}$ along a certain direction (*z*, e.g.) from the perturbation method

(50) $$v_{g,z}=-\frac{\text{Im}\left(ae^{i\frac{q_{z}a}{2}}{\tilde{u}}^{H}K_{R}\tilde{u}\right)}{2m\omega |\tilde{u}{|}^{2}}$$

to avoid the difficulty in implementing $\frac{\partial \omega }{\partial q}$ when phonon branches are heavily overlapped, which occurs often when the unit cell analysis contains many atoms.

The above dispersion analysis is based on a harmonic oscillation assumption. At temperatures beyond sub‐Kelvin, anharmonicity increases. In this case, the spectral energy density^[^ ^264^ ^]^ (SED) could be a better alternative for phonon dispersion relation calculation, especially in PnCs and MMs, since their unit cells usually contain a large number of atoms.

(51) $$\Phi (q,\omega )=\frac{1}{4\pi \tau_{0}N}\sum_{\alpha }\sum_{b}^{B}m|\int_{0}^{\tau_{0}}\sum_{l}^{N}v_{\alpha }\left(\begin{array}{l} l\\ b \end{array};t\right)\cdot e^{\left[iqx\left(\begin{array}{l} l\\ 0 \end{array}\right)-i\omega t\right]}dt{|}^{2}$$

where $\tau_{0}$ is the integration time constant, index *α* represents Cartesian coordinates, $v_{\alpha }\left(\begin{array}{l} l\\ b \end{array};t\right)$ is the *α* component of the instantaneous velocity of atom *b* with mass *m* inside unite cell *l*, *B* is the number of atoms in a unit cell, and *N* is the number of unit cells in the simulation domain.

Not only can SED reflect harmonic responses at elevated temperatures, but its relative amplitude provides another important anharmonic characteristic of phonon transport, phonon lifetime, *τ*, which can be fit with a Lorentzian function.

(52) $$\Phi (q,\omega )=\frac{I}{1+{\left[\frac{\omega -\omega_{c}}{\gamma }\right]}^{2}}$$

where *I* is the peak magnitude of the SED curve at a specified ***q*** and *ω*, $\omega_{c}$ is the frequency at the peak center, and *γ* is the half‐width at half maximum. Then the relaxation time τ=1/2γ.

Another phonon analysis usually performed for the derivation of thermal conductivity from Equation (32) and to understand the phonon frequency distribution, which may reveal the underlying phonon‐scattering mechanisms, is the phonon density of states (PDOS), like the electronic density of states, which describes the distribution of phonon modes over frequency *ω*, which is also introduced in the study by Parlinski et al.^[^ ^263^ ^]^

#### 3.2.4 Phonon Manipulation in Nanoscale Phononic Crystals and Metamaterials

Thermal management has always been an important topic in systems on a chip due to the performance sensitivity to heat in these miniature systems. Various phonon engineering mechanisms have been proposed at the nanoscale, such as reducing thermal transport by scaling down to nanowires^[^ ^265^ ^]^ to enhance phonon boundary scattering, introducing impurities, nanoparticles, voids, defects, and dislocations to increase the phonon impurity scattering rates,^[^ ^266^, ^267^ ^]^ and using mechanical strains and deformation to magnify phonon scattering due to anharmonicity.^[^ ^212^, ^242^, ^268^, ^269^, ^270^, ^271^ ^]^ The experimental realization of these mechanisms, however, requires high‐precision control to avoid interference of multiple effects. For example, in our previous study, although predictable and controllable thermal conductivity reduction in nanowires can be achieved via screw dislocation, which enhances anharmonic phonon scattering along the screw dislocation line, defects and surface modulation can further reduce their thermal conductivity due to the interference of the phonon defect and phonon surface scattering.^[^ ^267^ ^]^

**Figure 9:**
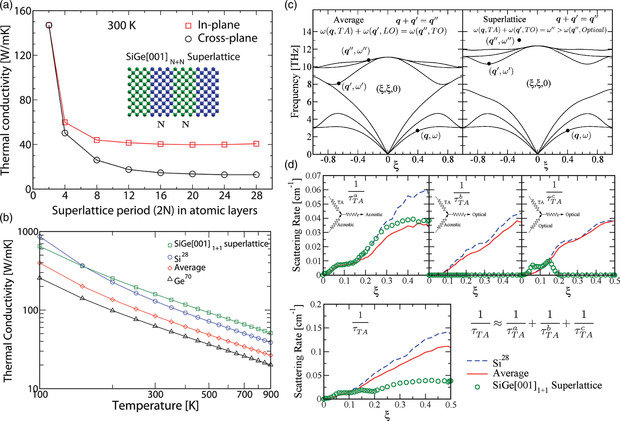
a) Variation of the computed *κ* of SiGe[001]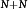 superlattices with superlattice period 2N. b) Comparison between the *κ* of pure Si, Ge, “average material” with effective Si‐Ge properties, and SiGe[001]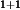. c) Comparison of phonon dispersions along (*ζ*,*ζ*,0) in an “average material” (left) and SiGe[001]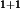 superlattice. d) Scattering rate of TA modes at 300 K due to various three‐phonon anharmonic scattering processes. a–d) Reproduced with permission.^[^ ^257^ ^]^ Copyright 2011, American Chemical Society.

On the other hand, superlattices have been extensively proposed to control phonon transport,^[^ ^1^, ^231^, ^237^, ^257^, ^272^, ^273^, ^274^ ^]^ in which the length of periodicity, *L*, is a critical parameter in tuning thermal conductivity. When *L* is much greater than the phonon MFP, phonons are insensitive to lattice periodicity, resulting in a diminished interference effect, and phonons can be considered as particles. On the contrary, when *L* is shorter than the intrinsic MFP of the constituent material, the MFP of the superlattice is limited by *L*, and phonons can be modeled as waves,^[^ ^1^ ^]^ which can be scattered at the interface, resulting in reduced *κ*. However, when *L* is further lowered, phonon bands are less folded, leading to a less reduced group velocity. The effect of group velocity is then competing against the thermal boundary resistance, leading to an increase in *κ* as *L* gets smaller.^[^ ^1^ ^]^ Moreover, ab initio calculations demonstrate that when *L* is on the order of a lattice parameter, *κ* of the superlattice can even exceed that of the constituent material,^[^ ^257^ ^]^ as shown in **Figure** **9**a,b. The phonon dispersion of such a superlattice resembles that of a PnC at the micro‐ and macroscales, in which bandgaps are created due to lattice mismatch. In contrast to bandgaps in their larger‐scale counterparts, where phonons are prevented from propagation in the bandgap frequency range, at the nanoscale, such a bandgap can instead promote phonon transport by reducing the three‐phonon anharmonic scattering rates, especially the acoustic + acoustic → optical and acoustic + optical → optical scattering channels, as shown in Figure 9. Although it has not been realized in nonlinear macro‐ and microscale PnCs, such a scattering mechanism might also inspire interesting phonon manipulation mechanisms at a higher length scales. It is worth noting that such three‐phonon processes mainly scatter low‐frequency phonons, which are not affected by interface roughness due to their long wavelengths. High‐frequency phonons, on the other hand, can be scattered by the interfacial disorder, which contributes to the overall *κ* reduction in super lattices.^[^ ^231^ ^]^

**Figure 10:**
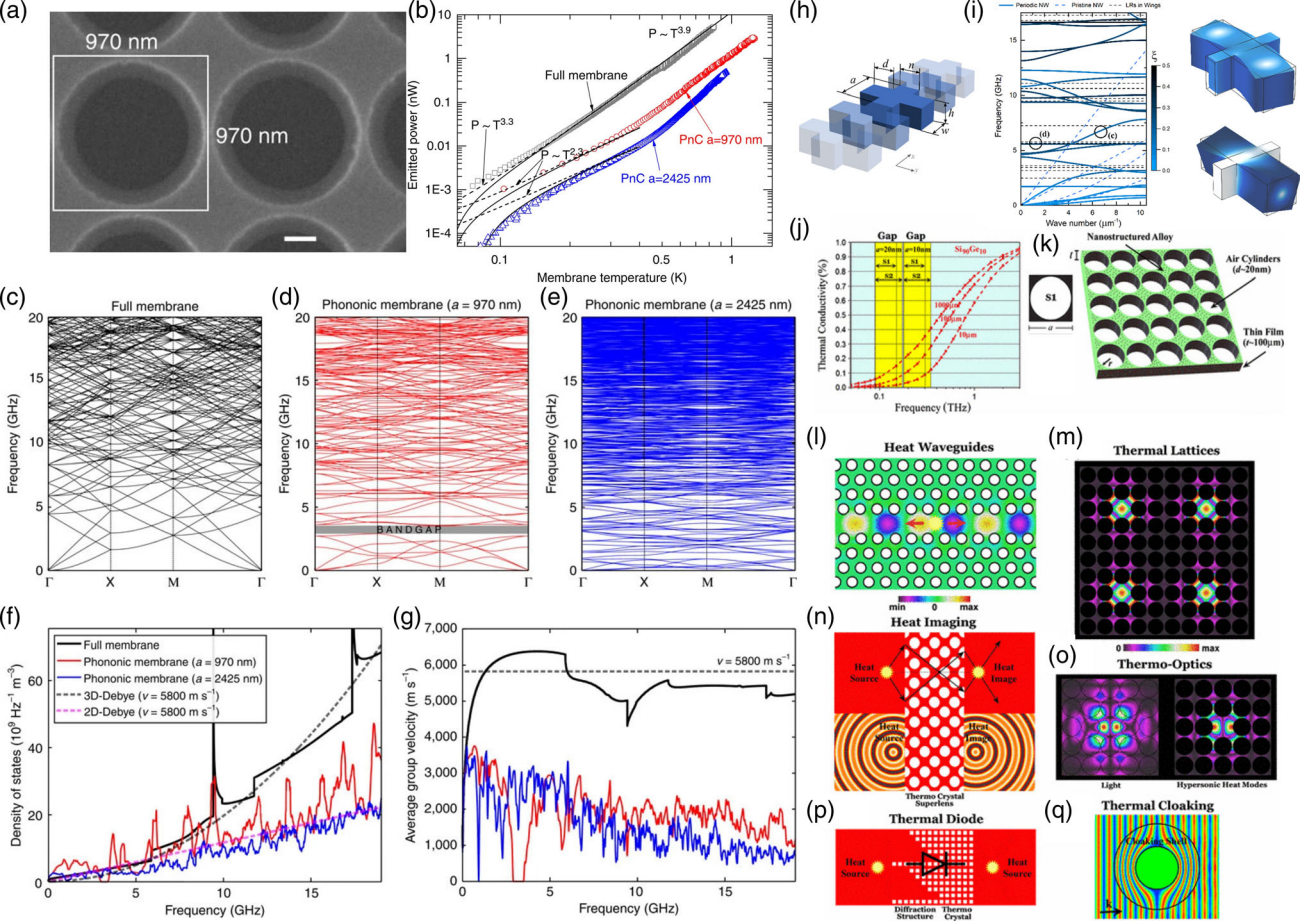
a) SEM image of a region of a PnC sample (black area: empty space, grey area: SiN membrane), showing the unit cell size 970 nm×970 nm and the width of the narrowest region of ≈60 nm. Scale bar: 200 nm. b) Measured emitted phonon power versus temperature. c–e) Phonon dispersion relations of a full membrane (c) and square lattices with cell size equal to 970 nm (d) and 2425 nm (e). f) PDOS and g) average group velocity of the membrane and PnCs are calculated using the whole reciprocal space. a–g) Reproduced with permission.^[^ ^224^ ^]^ Copyright 2104, The Authors, published by Springer Nature. h) Schematic of a simulated Si nanowire with wings. i) Phonon dispersion of the nanowire with (solid) and without (dashed) wings. Colors of the curves indicate the physical location of the mode, with blue showing the modes inside the nanowire and black inside the wings, as shown in the mode shapes on the right. h,i) Reproduced under the terms of the CC‐BY Creative Commons Attribution 4.0 International license (https://creativecommons.org/licenses/by/4.0).^[^ ^228^ ^]^ Copyright 2019, The Authors, published by MDPI. j) The yellow areas show the frequency gaps corresponding to the films patterned with a square arrangement of air cylinders with the unit cell and full‐scale structure shown in (k). l–q) Proposed nano‐PnC applications as heat waveguides (l), defect‐induced localized hypersonic heat modes (m), a heat image superlens (*n*), point‐defect‐induced localization of both light and hypersonic heat modes (o), thermal diodes (p), and thermal cloaking (q). j–q) Reproduced with permission.^[^ ^226^ ^]^ Copyright 2013, American Physical Society.

The coherence of long‐wavelength phonons can be interfered by introducing perturbations varying over long length scales,^[^ ^231^ ^]^ making it suitable for in situ coherent phonon manipulation via nano‐PnCs and MMs. Due to the experimental limitation on the unit cell sizes of nano‐PnCs and MMs, many such studies were conducted at low temperatures so that wavelengths can surpass unit cell sizes.^[^ ^224^, ^226^, ^228^, ^229^, ^275^ ^]^ For example, as shown in **Figure** **10**a,b, an increased PnC unit cell size can reduce *κ*.^[^ ^224^ ^]^ This is because, as unit cell size increases, more bands are folded into the Brillouin zone, reducing PDOS and group velocities of coherent phonons, as shown in Figure 10c–g. Note that, although a bandgap is generated in the PnC with a smaller unit cell, the main influence of the thermal transport suppression is due to a combination of the reduced PDOS and group velocities.^[^ ^224^ ^]^ As temperature is elevated, phonon transport transitions from coherent to incoherent as high temperatures excite high‐frequency phonons, which have higher diffusive scattering probabilities than low‐frequency ones, making it difficult to manipulate phonon transport with PnCs with large periodicity.^[^ ^275^ ^]^ Similarly, coherent phonons can also be localized in nanoscale MMs with periodic resonators,^[^ ^228^ ^]^ as shown in Figure 10h,i. Since coherent phonons transport can be easily predicted by phonon dispersion relations, many interesting applications can be achievable in nanoscale PnCs and MMs. A more comprehensive review on phonon coherence can be found in the study by Xie et al.^[^ ^276^ ^]^

**Figure 11:**
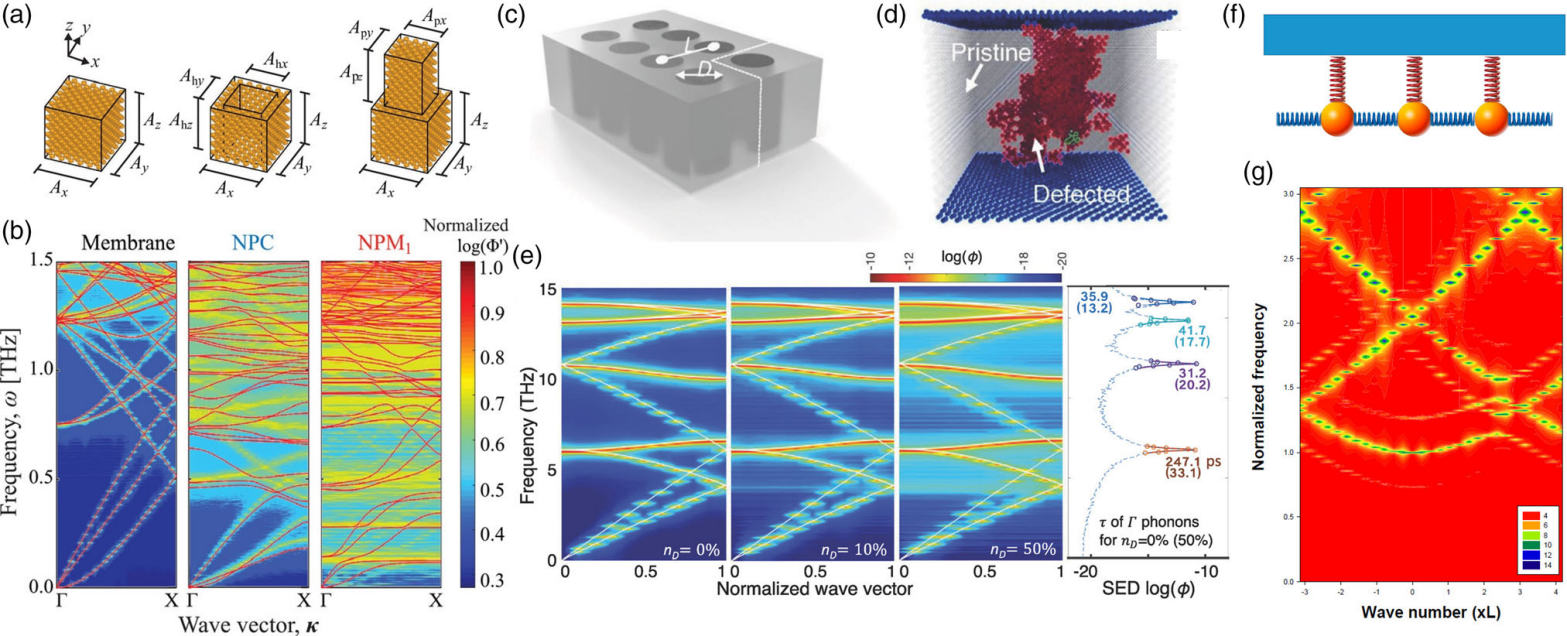
a) Atomistic‐scale unit cells of a pristine membrane, a membrane PnC with square‐shaped holes (NPC), and a membrane MM with periodic resonators (NPM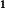). b) Phonon dispersion relations of the pristine membrane, NPC and NPM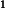, corresponding to (a). The solid lines represent the dispersion curves obtained from LD calculations. The color contours represent the SED spectra, which are each normalized by the corresponding maximum value. a,b) Reproduced with permission.^[^ ^277^ ^]^ Copyright 2020, Wiley‐VCH. c) Schematic of hybrid ordered/discorded silicon, where dark areas denote damaged domains patterned by ion beam irradiation. d) Atomistic view of the damaged region. e) SED for patterned silicon nanocrystals with 0%, 10%, and 50% damage in the defective region, respectively. Phonon lifetimes of the structures fit with the Lorentzian functions at the Γ point with 0% and 50% damage are calculated and presented on the right. c–e) Reproduced with permission.^[^ ^278^ ^]^ Copyright 2018, Wiley‐VCH. f) Schematic illustration of the harmonic chain grounded to a substrate via side springs. g) Phonon dispersion calculated with SED normalized to the lowest value of the unperturbed band. The brighter branches correspond to the usual zeroth‐order type wave, while the fainter ones parallel to the brighter ones are characteristic of first‐order waves. f,g) Reproduced under the terms of the CC‐BY Creative Commons Attribution 4.0 International license (https://creativecommons.org/licenses/by/4.0).^[^ ^279^ ^]^ Copyright 2016, The Authors, published by MDPI.

Although the characterization of coherent phonons requires wavelengths longer than the periodicity and low operating temperature,^[^ ^226^ ^]^ as long‐wavelength phonons are largely destroyed, they are not completely annihilated at room temperature, as was observed by Alaie et al.^[^ ^255^ ^]^ However, at high temperatures, the effect of anharmonic phonon scattering becomes important, leading to a modified phonon dispersion relation with broadened phonon bands, obstructing phonon transport. Phonon dispersion at higher temperatures can then be calculated with SED to account for anharmonicity.^[^ ^277^, ^278^, ^279^, ^280^ ^]^ For example, inspired by PnCs and AMMs at the continuum scale, Hussein et al.^[^ ^277^ ^]^ applied SED to analyze phonon transport of silicon membrane without (pristine) and with periodic voids as nano‐PnCs (to be consistent with the referenced paper, we use NPC as abbreviation here) and resonators as nanophononic metamaterials (to be consistent with the referenced paper, we use NPM as abbreviation here), as shown in **Figure** **11**a,b. Phonon dispersion relations obtained from SED agree well with that calculated by LD for the pristine membrane at the low‐frequency regime (below 1.2 THz), indicating the relatively weak anharmonicity at room temperature for the studied systems. Evidently, the NPC phonon branches are more flattened due to Bragg scattering and horizontal bands appear at the low‐frequency regime in NPM due to local resonance, suggesting a significant reduction in group velocities of both structures, although via different mechanisms, leading to a reduction in their overall thermal conductivity. Additionally, resonators in NPM can trap propagating phonons, leading to more significant thermal conductivity reduction with higher resonating pillars.

Zhu et al.^[^ ^278^ ^]^ proposed using ion beam irradiation to damage crystalline silicon to create PnCs with ordered/disordered nanocomposites, as shown in Figure 11c,d, to modulate phonon transport. They found that with an increased damage ratio, the peaks of SED are more broadened, resulting in diminished phonon lifetimes fit with Equation (52), as shown in Figure 11e, which partly contributes to a reduction in the thermal conductivity of PnCs with defects. In addition, using MD simulations, Ren et al.^[^ ^281^ ^]^ acquired a remarkable reduction of interfacial thermal resistance across the interface between pristine black phosphorene and its PnC (with periodic perforations) compared with those in mass‐mismatched interfaces. Moreover, in contrast to conventional heterostructures with dissimilar materials, the thermal conductance across such a phononic‐mismatched heterostructure has a weak temperature dependence and thermal rectification, suggesting a new paradigm in phonon transport manipulation via nano‐PnCs. These findings suggest that phonons transport can be manipulated by PnCs and MMs due to both the alteration of the dispersion relation, which changes the group velocity, but also enhances anharmonic scattering that reduces a majority of the phonon lifetimes. Phononic MMs can also trap phonons in resonators, adding additional thermal conductivity lowering pathway.

In terms of the application of SED in PnC and MM studies, it is also worth noting that, although SED‐derived phonon dispersion is usually analyzed to understand thermal transport in nanomaterials, it can also be applied in PnCs and AMMs in general. For example, Deymier and Runge^[^ ^279^ ^]^ calculated the phonon dispersion relations using SED for a mass‐spring system, shown in Figure 11f, and detected band shifts due to first‐order particular solutions (the first‐order waves are with orbital and spinor parts). The spatial modulation causes the bands to fold, enabling overlap and hybridization between the frequency‐shifted Bloch modes and the original Bloch modes of the lattice, which open bandgaps in a band structure that has lost its mirror symmetry at the origin of the Brillouin zone. Such bandgap creation due to spin–orbit manipulation of the elastic system can inspire the construction of a more intrinsically unconventional topology in PnCs and AMMs.

## 4 Micro‐ and Nanoscale Topological Phononic Crystals and Metamaterials

In recent years, the advent of topological PnCs and MMs has emerged and attracted significant attention in the application of phonon manipulation. The concept of topological insulators (TIs) originates from quantum physics,^[^ ^282^ ^]^ in which a topological invariant is often used to describe a category of materials or structures that present the same types of confined conductive states. Take Chern insulators as an example. Once the Dirac point is identified, a Berry phase (or Berry flux) Φ is calculated by

(53) Φ=∮dk×A

where $A=-i\langle \psi (k)|\nabla_{k}|\psi (k)\rangle$ is the Berry connection, which can be obtained from the eigenvectors of a phonon band |ψ(k)⟩. The Chern number, *C*, can then be obtained from

(54) $$C=\frac{1}{2\pi }\iint_{BZ}\nabla \times Ad^{2}k$$

where BZ denotes the Brillouin zone, and ∇×A is typically referred to as the Berry curvature. To obtain nontrivial topological properties, such as the topological edge/surface states and higher‐order topological corner states (HOTI), *C* must be nonzero, which means a flux of properties must go through the Berry curvature. Thus, when a bulk bandgap and a nonzero *C* coexist, a topological phase transition emerges from the insulating state to the conductive state with confined gapless states.

A flux of mechanical analogs of quantum topological insulating phenomena have emerged in the past decade, including the quantum Hall effect (QHE), which allows one‐way, nonreciprocal wave propagation by creating chiral edge states that are robust against defects and disorders^[^ ^283^, ^284^, ^285^, ^286^ ^]^ by breaking time‐reversal symmetry through the application of an external field. Meanwhile, the realizations of the quantum spin Hall effect (QSHE)^[^ ^287^, ^288^, ^289^ ^]^ and quantum valley Hall effect (QVHE)^[^ ^290^, ^291^, ^292^, ^293^, ^294^, ^295^, ^296^, ^297^ ^]^ have also been achieved in fully passive PnCs and AMMs to guide phononic waves to propagate along the topologically protected edges at the bandgap frequency, while being immune to backscattering at corners or defects. Very recently, higher‐order TIs (HOTI)^[^ ^298^, ^299^, ^300^, ^301^, ^302^, ^303^, ^304^, ^305^, ^306^, ^307^ ^]^ emphasizing on highly localized topological corner states have attracted great attention in the PnC and AMM communities. In addition, mechanical systems involving topologically protected floppy edge states (TPFES)^[^ ^40^, ^187^, ^308^, ^309^, ^310^, ^311^, ^312^, ^313^ ^]^ have also been proposed theoretically and realized experimentally to localize waves on certain edges. As in the case of quantum Hall‐derived examples, these TPFES are also intrinsic features manifested on the edges due to the unit cell topology that governs their bulk characteristics. More thorough reviews on phononic TIs can be found in other studies.^[^ ^29^, ^30^, ^31^, ^32^, ^33^ ^]^

As discussed earlier, the wave‐guiding and wave localization capabilities are intrinsic to the topology of TIs; thus, they are more advantageous in applications compared with conventional PnCs and AMMs, the latter of which are more prone to perturbations and defects. Despite a large number of research efforts devoted to TI studies, very few attempted to realize them at the micro‐ or nanoscales, despite the importance of GHz–THz signal sensing and processing in modern electronics applications. Here, the sporadic existing examples that have investigated TIs at these two levels are reviewed.

**Figure 12:**
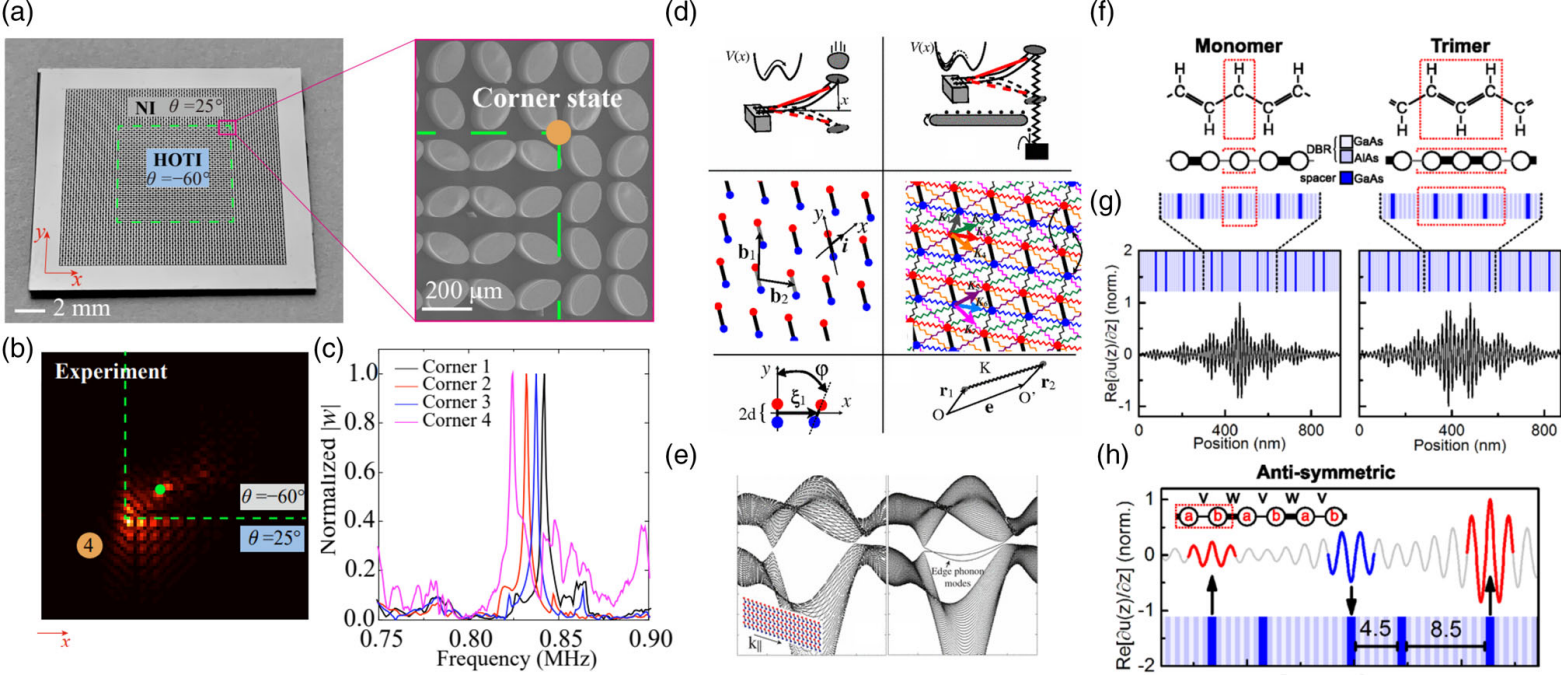
a) Mechanical HOTI on a silicon chip. b) Experimentally measured displacement profile at the corner. c) Measured source‐to‐detector spectra for the four corners. a–c) Reproduced with permission.^[^ ^314^ ^]^ Copyright 2021, Elsevier. d) A 2D tubulin sheet modeled as 2D lattices of rigid dimers with harmonic interactions. e) Phonon dispersion relation of a simulated 2D microtubule sheet with periodic (left) and open (right) boundary conditions. d,e) Reproduced with permission.^[^ ^315^ ^]^ Copyright 2009, American Physical Society. f) Topologically trivial (left) and nontrivial (right) phases of the SSH model with corresponding polyacetylene molecules. g) Strain distribution of topological monomer (left) and trimer(right) defect states in coupled phononic cavities. h) Antisymmetric topological edge states in nanophononic cavity array. Red and blue curves indicate alternating signs of the strain. f–h) Reproduced with permission.^[^ ^316^ ^]^ Copyright 2018, American Physical Society.

Wu et al.^[^ ^314^ ^]^ implemented the HOTIs by etching patterns on a silicon chip and have successfully observed the topologically protected corner states at four corners within the bulk/edge bandgap frequency range, as shown in **Figure** **12**a–c. Although a similar system has been achieved in a bigger structure before,^[^ ^306^ ^]^ the on‐chip study confirmed the scalability of these topological states and promises their feasibility in MEMS for vibration isolation or energy‐harvesting applications.

At the nanoscale, much of the effort is theoretical. The complexity of atomic/molecular interactions poses not only great challenges to experimental characterization but also demands highly idealized scenarios in theoretical models. For example, Prodan and Prodan^[^ ^315^ ^]^ concluded the existence of topologically protected edge states (TPES) in microtubules by modeling the tubulin sheet as a 2D lattice of rigid dimers with harmonic interactions, as shown in Figure 12d. The authors also restrict the study of propagating oscillatory motions that involve displacements and rotations of the dimers in the fixed xy local planes with only two degrees of freedom. An asymmetry is then induced via a small difference in spring constants $K_{3}$ and $K_{6}$ in Figure 12d to open a Dirac bandgap. TPES can thus be achieved within this bandgap with a non‐zero Chern number, Figure 12e. Such TPES may be associated with the microtubules’ dynamic instability on the edge, which may cause the tubes to depolymerize. Another nanoscale study investigates the Zak phases in transpolyacetylene^[^ ^316^ ^]^ using the Su–Schrieffer–Heeger^[^ ^317^, ^318^ ^]^ (SSH) model and demonstrated symmetric and asymmetric TPES in nanophononic cavity array, as shown in Figure 12f–h. Although this research is highly valuable in understanding the acoustic states in TIs at the nanoscale using an example of a single straight polyacetylene chain, actual polyacetylene chains are usually highly entangled at room temperature and present high radii of gyration. The applicability of such an idealized nanoscale structure might be difficult to realize. Jiang et al.^[^ ^319^ ^]^ also investigated the existence of the QVHE in SiC and BN systems. They obtained their phonon dispersion relations using LD and achieved full‐scale simulation with MD simulations. However, the experimental realization of such a system requires ultralow temperatures and excitation with ultrahigh precision and an extremely narrow band to excite the edge modes. Also, the atomic interactions are usually highly nonlinear. Hence, the excited atom displacements should be maintained at a very low amplitude. As we can see, studies (both experimental and theoretical) of realistic phononic TIs are not trivial yet potentially offer many interesting opportunities for a broad range of quantum engineering, including phonon–photon and/or –electron coupling.^[^ ^320^, ^321^, ^322^, ^323^ ^]^

## 5 Conclusion

Fundamentals of wave manipulation mechanisms that are universally applicable at any length scale have been summarized. The most common numerical simulation used to aid the design of microscale PnCs and AMMs is the conventional finite‐element method. Although FEM is convenient and commercially available in software such as COMSOL Multiphysics, its accuracy largely depends on the element and time‐step sizes, which are directly correlated with the memory and computational time consumption. On the other hand, various other numerical approaches, including the transfer matrix method, the generalized differential quadrature method, and the generalized homogenization framework, among many others, have been developed, potentially with higher accuracy and efficiency, depending on the problems to solve. Thus, choosing the right computational model for theoretical prediction is the key to acquiring an accurate understanding of wave propagation. Moreover, an optimized topology searched with an appropriate topology optimization method, including the GTO method, GA, and ML, can help with the efficient design of structures with the most pronounced desirable properties, thus easing the burden on micro‐ and nanofabrication and characterization. In addition, both material and geometric nonlinearity can exist in microscale PnCs and AMMs due to their size and the choice of constituent materials. Thus, accurate modeling will help with the understanding of the nonlinear behaviors, which can help promote the application of nonlinearity in PnCs and AMMs to achieve more exotic properties, such as reconfigurable wave propagation, harmonic tuning, and nonreciprocal wave transport. However, prevalent applications of the above theoretical methods and analysis are yet to be widely applied in microscale PnC and AMM studies. Resorting to an accurate mathematical model, designing an optimized configuration, and acquiring a better understanding of the nonlinear behaviors of PnCs and AMMs are believed to be able to facilitate the advancement of experimental effort in micro‐ and nanofabrication, which will, in turn, deepen our understanding of the phononic behaviors.

For nanoscale PnCs and MMs, progress is still being made in understanding the thermal engineering mechanisms. A combination of theoretical prediction and experimental characterization will often work together to facilitate the understanding and engineering of phonon transport in PnCs and MMs. As discussed above, characterizing phonons in PnCs and MMs at this scale is not trivial. A good understanding of the phonon transport is needed prior to any experimental design, which is challenging due to the intrinsic complexity of phonon interactions with the boundaries, impurities, and other phonons. The inclusion of periodic patterns adds more complications. Similarly, apart from the experimental progress, an accurate numerical prediction which requires an appropriate analytical method, a good description of atomic interaction, sufficiently large simulated systems, and a high number of time steps to obtain reliable statistical averages are also of great importance in understanding phonon transport and interactions in nanostructures.

At the end, a new category of PnCs and AMMs, that is, TIs, has been reviewed. These PnCs and AMMs are more attractive due to their robustness against small perturbations and disorders, which is unachievable with conventional PnCs and AMMs. Although the study of such PnCs and AMMs at the microscale has just started, the level of difficulty in theoretical analysis and experimental realization is not significantly higher than the conventional ones at this scale. Hence, studies on the microscale TIs can be expected to flourish shortly. On the other hand, obstacles in theoretical modeling of nanoscale TIs persist due to complex atomistic network and strong nonlinearity. The few existing studies reviewed in Section 4 are all limited to highly simplified models. More realistic analytical methodologies that can identify or decouple certain topological phonons in complex nanoscale dynamic systems are yet to be developed. The reliability of such analytical methodologies may be verified with advanced phonon characterization methods, such as neutron scattering.^[^ ^324^, ^325^, ^326^ ^]^

Despite the scarcity of micro‐ and nanoscale phonon engineering using PnCs and MMs due to challenges in both experimental realization and numerical prediction, the efforts are worth taking. Recent studies have shown that phonons may also serve as important media in quantum information transfer and distribution^[^ ^327^, ^328^, ^329^ ^]^ like electrons and photons. Thus, understanding and manipulating phonon transport is not only helpful to well‐explored acoustic sensing and thermal management applications but will also have the potential to advance quantum information processing technologies, such as quantum computing.

## Conflict of Interest

The author declares no conflict of interest.
